# Pharmacological Suppression of CNS Scarring by Deferoxamine Reduces Lesion Volume and Increases Regeneration in an *In Vitro* Model for Astroglial-Fibrotic Scarring and in Rat Spinal Cord Injury *In Vivo*

**DOI:** 10.1371/journal.pone.0134371

**Published:** 2015-07-29

**Authors:** Christina Francisca Vogelaar, Brigitte König, Stefanie Krafft, Veronica Estrada, Nicole Brazda, Brigida Ziegler, Andreas Faissner, Hans Werner Müller

**Affiliations:** 1 Molecular Neurobiology Laboratory, Department of Neurology, Heinrich-Heine-University of Duesseldorf, Duesseldorf, Germany; 2 Institute of Microanatomy and Neurobiology, Johannes Gutenberg-University Mainz, Mainz, Germany; 3 Institute for Cellular Morphology and Molecular Neurobiology, Ruhr-University Bochum, Bochum, Germany; Hertie Institute for Clinical Brain Research, University of Tuebingen., GERMANY

## Abstract

Lesion-induced scarring is a major impediment for regeneration of injured axons in the central nervous system (CNS). The collagen-rich glial-fibrous scar contains numerous axon growth inhibitory factors forming a regeneration-barrier for axons. We demonstrated previously that the combination of the iron chelator 2,2’-bipyridine-5,5’-decarboxylic acid (BPY-DCA) and 8-Br-cyclic AMP (cAMP) inhibits scar formation and collagen deposition, leading to enhanced axon regeneration and partial functional recovery after spinal cord injury. While BPY-DCA is not a clinical drug, the clinically approved iron chelator deferoxamine mesylate (DFO) may be a suitable alternative for anti-scarring treatment (AST). In order to prove the scar-suppressing efficacy of DFO we modified a recently published *in vitro* model for CNS scarring. The model comprises a co-culture system of cerebral astrocytes and meningeal fibroblasts, which form scar-like clusters when stimulated with transforming growth factor-β (TGF-β). We studied the mechanisms of TGF-β-induced CNS scarring and compared the efficiency of different putative pharmacological scar-reducing treatments, including BPY-DCA, DFO and cAMP as well as combinations thereof. We observed modulation of TGF-β-induced scarring at the level of fibroblast proliferation and contraction as well as specific changes in the expression of extracellular matrix molecules and axon growth inhibitory proteins. The individual and combinatorial pharmacological treatments had distinct effects on the cellular and molecular aspects of *in vitro* scarring. DFO could be identified as a putative anti-scarring treatment for CNS trauma. We subsequently validated this by local application of DFO to a dorsal hemisection in the rat thoracic spinal cord. DFO treatment led to significant reduction of scarring, slightly increased regeneration of corticospinal tract as well as ascending CGRP-positive axons and moderately improved locomotion. We conclude that the *in vitro *model for CNS scarring is suitable for efficient pre-screening and identification of putative scar-suppressing agents prior to *in vivo* application and validation, thus saving costs, time and laboratory animals.

## Introduction

After traumatic spinal cord injuries meningeal fibroblasts invade the lesion site, where they form a fibrous scar. Subsequently, activated astrocytes start surrounding the fibrous lesion core and create a glia limitans to protect the nervous tissue from the external environment and restore the blood-brain-barrier. The glial and fibrous compartments of the scar each contain different types of extracellular matrix (ECM) and axon growth inhibitory molecules that are differentially regulated over time [1–4]. The astroglial outer region of the scar is marked by the astrocytic glial fibrillary acidic protein (GFAP) and contains numerous chondroitin sulphate proteoglycans (CSPGs) among which are NG-2, neurocan, and phosphacan. The central fibrous region is marked by fibronectin, due to invading meningeal fibroblasts, and contains Tenascin C (Tnc) and NG-2 [1–3], Semaphorin 3A (Sema3A) [5], Ephs and Ephrins [6]. Most of these inhibitory molecules peak at 1–2 weeks after injury. Over longer periods of time (3 to 12 months), the fibrous region decreases in size due to a contraction of the connective tissue matrix [7].

We and others showed that after spinal cord injury (SCI) transected axons that try to regenerate are able to penetrate the reactive glial compartments but stop at the fibrous core [1, 8]. We postulated that soluble inhibitory factors might bind a scaffold of extracellular matrix molecules in the fibrous scar [4]. In previous *in vivo* experiments in rats we found that reducing the formation of the fibrotic scar by preventing the deposition of collagen IV during 1–2 weeks decreased the levels of NG-2 and led to enhanced axon regeneration of various types of axon tracts through the scar [8–10]. This so called anti-scarring treatment (AST) consisted of the iron chelator 2,2’-bipyridine-5,5’-decarboxylic acid (BPY-DCA) and the signaling molecule cyclic AMP (cAMP). Iron chelators interfere with the synthesis of collagen by depriving the enzyme prolyl-4-hydroxylase of its cofactor iron [11]. Cyclic AMP on the other hand is known to inhibit fibroblast proliferation and collagen biosynthesis [12]. AST treatment resulted in enhanced functional recovery of rats that received a dorsal hemisection of the spinal cord at level T8 [8]. Although we observed a reduction in collagen IV and NG-2 immunofluorescence after AST, the mechanism of lesion scarring, scar suppression and axon growth inhibition of the scar remained unclear. Therefore, in the present study we aimed to use an *in vitro* model to study the mechanisms of scar formation and reduction as well as to provide an assay system to investigate new scar-reducing treatments.

Most *in vitro* models for scar formation are axon crossing border assays. Often, stripes or gradients of permissive and inhibitory molecules, e.g. laminin and CSPG [13, 14] or membrane preparations of inhibitory cells [15] are used. In other studies, cell types that are permissive or inhibitory for axon growth are mixed, like Schwann cells and astrocytes [16] or meningeal fibroblasts and astrocytes [17]. These models, however, lack the 3-dimensional characteristics of a dense fibrous scar. In a recent model astrocytes and fibroblasts were plated on culture inserts and lesioned by applying pressure [18]. In 2010, Kimura-Kuroda et al introduced a three-dimensional model, in which scar-like cell clusters are formed [19]. In this model, astrocyte and fibroblast monolayers are cultured in close proximity and stimulated with transforming growth factor-beta 1 (TGF-β1). In response to TGF-β1, the fibroblasts form clusters, which, at the astrocyte-fibroblast border, are surrounded by astrocytes, thus resembling the *in vivo* scar compartments in traumatic spinal cord lesions. Indeed, TGF-β1 is well-known for its role in wound healing and fibrosis in many body tissues [20–22]. It is also firmly established that TGF-β1 is upregulated after spinal cord injury [23–26]. TGF-β receptors are present on meningeal fibroblasts invading the lesion site [27]. TGF-β also induces the expression of CSPGs and Tnc in astrocytes [28–30]. In fact, several *in vivo* studies have already confirmed that the inhibition of TGF-β1 by neutralizing antibodies promotes axon regrowth and functional recovery after spinal cord contusion injury [31, 32]. However, there are also reports about positive effects of TGF-β1 on neuroprotection and neuropathic pain [33, 34]. One study using TGF-β1 neutralizing antibodies suggested enhanced cavitation, due to the inhibition of its anti-inflammatory properties [35] and another showed a reduction of lesion volume after treatment with TGF, probably due to reduced inflammation [36]. Finally, a study in the lab of James Fawcett clearly showed that TGF-β antibodies can indeed reduce the scar without increasing axon regeneration [37].

The above studies suggest that TGF-β1 has positive effects via its anti-inflammatory properties and negative effects via fibrous scarring. Treatments for spinal cord injury should, therefore, reduce the effects of TGF-β1 on invading meningeal fibroblasts. Interestingly, a recent study suggests that TGF-β1 influences the iron-homeostasis of astrocytes and microglia [38], causing iron efflux from the astrocytes and iron retention in the microglia.

Here, we adapted the meningeal fibroblast/astroglial co-culture model of TGF-β-induced fibrous scarring of Kimura-Kuroda et al (2010) [19] to investigate both the mechanisms of scar formation and the scar-reducing properties of iron chelators and cAMP. In particular, we studied the influence of the individual and combinatorial treatments on the number of scar-like cell clusters and their composition as well as on neurite outgrowth of neonatal cortical neurons seeded onto the clusters. The iron chelator deferoxamine (DFO) proved to be the most effective treatment to suppress scar-like cluster formation resulting in the reduction of both the scar area and its inhibitory properties as well as to increase the neurite length of the cortical neurons on the remaining clusters. In parallel, we investigated and compared the scar-reducing properties of DFO, BPY-DCA and cAMP in the *in vivo* dorsal hemisection SCI model in rats and observed that all three individual treatments were able to reduce collagenous scarring when applied by local intrathecal infusion over 7 days. DFO was further tested on axonal and functional regeneration over a long-term period of 19 weeks. Infusion of DFO during the first 14 days post lesion led to a reduction of the scar size and preservation of spinal cord tissue. A slight increase in corticospinal tract and ascending CGRP tract regeneration was observed, as well as a moderate improvement of locomotor behavior, indicating partial functional recovery.

## Materials and Methods

### Animal experiments

All experiments on laboratory rats were in compliance with the German Animal Protection Law (State Office, Environmental and Consumer Protection of North-Rhine-Westphalia, LANUV NRW, Recklinghausen, Germany). The experiments were approved by LANUV NRW under Az: 8.87–50.10.34.09.081 and 8751.04.2011.A023 LANUV NRW is the Institutional Animal Care and Use Committee in the State of Northrhine-Westphalia. The experiments were performed according to the "Guidelines for good scientific practice at the Heinrich-Heine-University Düsseldorf" decreed by the Senate of the Heinrich-Heine-University Düsseldorf and approved by Dr. M. Sager, animal welfare officer of the Heinrich-Heine-University Düsseldorf. Experimental animals were housed in groups under standard conditions. Water and food were available *ad libitum*.

### Isolation of cortical astrocytes, meningeal fibroblasts and cortical neurons

All cell types were derived from P0-2 neonatal Wistar rats. The pups were killed using isoflurane and decapitation. The brain was dissected and primary cortical astrocytes were isolated as described previously [39]. Primary meningeal fibroblasts were isolated from the meninges by incubation with 0.05% trypsin/EDTA (Invitrogen) for 45 min at 37°C. After stopping the reaction with DMEM/FBS 10% the cells were triturated, resuspended in medium and filtered through 60 μm nylon mesh. The fibroblasts were then propagated in DMEM glutaMAX (Invitrogen) containing 10% FBS and 50 Units penicillin/streptomycin (P/S, Invitrogen). Primary cortical neurons were isolated from neonatal cortex using the methods previously described for embryonic cortical neurons [40].

### *In vitro* scarring model

**Fig 1:**
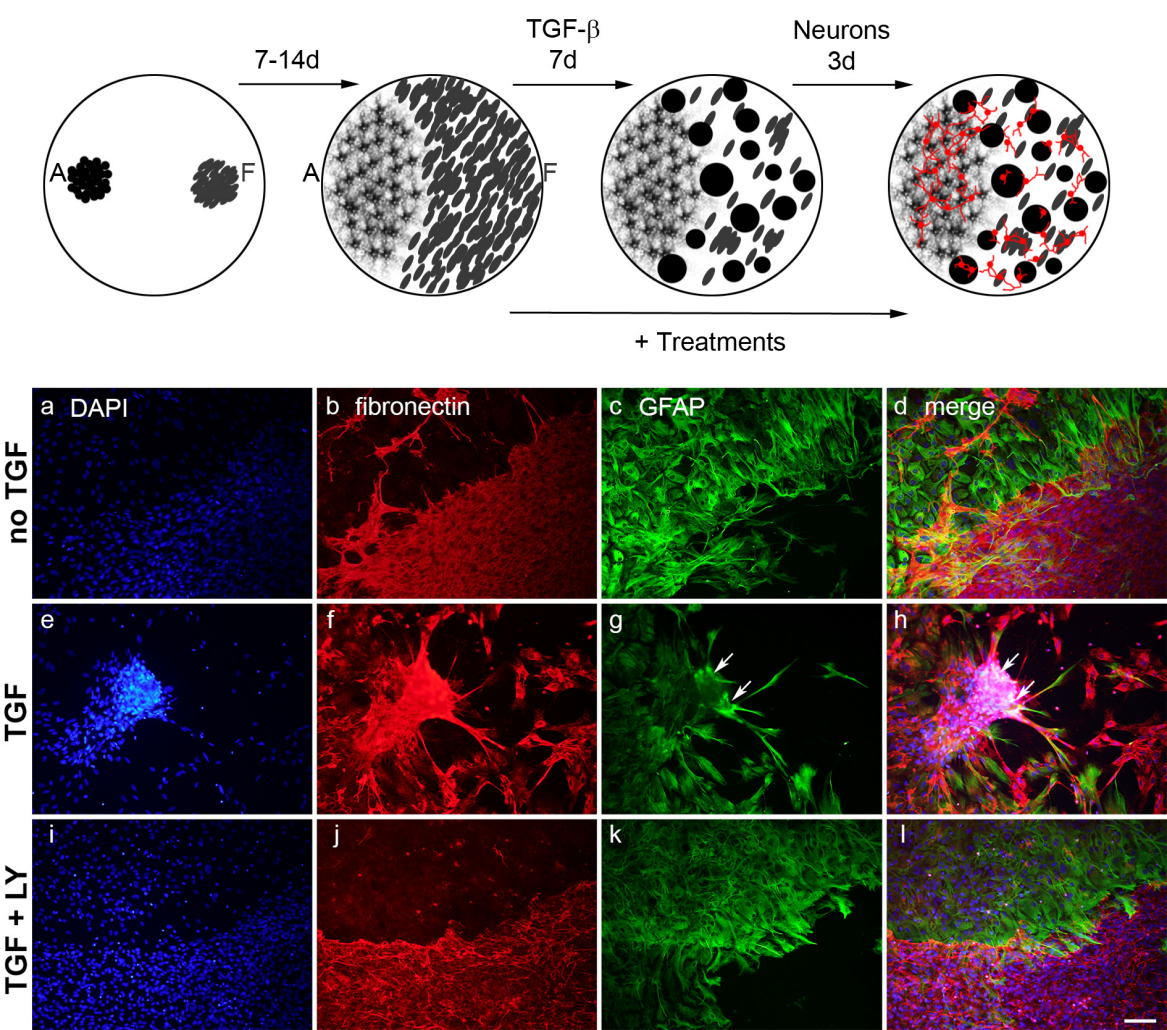
Schematic overview of the *in vitro* scarring model. Droplets of cortical astrocytes (A) and meningeal fibroblasts (F) were plated and allowed to grow in monolayers that contacted each other between 7 and 14 days. Then, TGF-β1 was added and incubated for 7 d. During this period, clusters were formed by the fibroblasts and those that appeared at the fibroblast to astrocyte border were surrounded by astrocytes. At 7 d after TGF-β-stimulation, dissociated neonatal cortical neurons (in red) were plated and allowed to grow for another 3 d. Potential scar-reducing treatments were applied starting from the time point of TGF stimulation. (a-d) Immunocytochemical staining for fibronectin (red), GFAP (green) and DAPI (blue) showing no cluster formation in astrocyte–fibroblast co-cultures without TGF-β. (e-h) TGF-β induced cluster formation. Clusters formed at the border of the two cell types consisted of meningeal fibroblasts surrounded by astrocytes (arrows). (i-l) Cluster formation was abolished by the TGF-β inhibitor LY364947. Scale bar = 100 μm.

After propagation of the cortical astrocytes and meningeal fibroblasts (maximally 1 and 5 passages respectively) the cells were deposited as droplets containing 15,000 cells each (Fig 1) onto coverslips coated with 0.1 mg/ml poly-D lysine (Sigma). These were allowed to settle for max. 5 h after which they received GlutaMAX medium containing 10% FBS, 2 mM L-glutamine and 50 Units P/S. The co-cultures were incubated for 10–16 d in a 37°C incubator under 10% CO_2_ until the layers were confluent and contacted each other. Subsequently, 10 ng/ml of recombinant human TGF-β1 (R&D Systems) was added to the medium and incubated for 7 d, inducing scar-like cluster formation. At 7 d after the treatment of the co-cultures with TGF-β1 50,000 cortical neurons per well were added to the culture medium and incubated for 3 more days. Medium was refreshed 1 d prior to plating of the neurons, so that the astrocytes had sufficient time to condition the medium for axon growth.

### Scar-reducing treatments

Treatments were applied simultaneously with TGF stimulation. The iron chelators deferoxamine mesylate (DFO, Novartis) and 2,2’-bipyridine-5,5’-decarboxylic acid (BPY-DCA, Sigma) were used at 10 and 50 μM respectively. A 100x stock solution of 5 mM BPY-DCA was dissolved in 20 mM Tris, therefore, a 200 μM Tris concentration was used as vehicle control. In addition, 8-Br-cyclic AMP (cAMP, Sigma) was used at a final concentration of 0.5 mM either alone or in combination with each of the iron chelators. DFO and cAMP were dissolved in H_2_O, therefore, similar volumes of H_2_O were used as controls. The treated co-cultures were fixed at 7 d after treatment (including a refreshment of medium, TGF and treatments at day 4) or co-cultured for 3 more days with neonatal cortical neurons as described above. A total of 6 coverslips was analyzed for the scar-reducing capacities of the treatments. The number of clusters per coverslip was counted and their size was measured using Image J.

### Proliferation and live cell imaging

For proliferation assays, bromodeoxyuridine (BrdU) was added at the time of TGF-β stimulation and cultures were fixed after 6 and 24 h, respectively. For live cell imaging, the cultures were incubated with TGF-β1 in the above described medium for 10–24 hours, then the medium was changed to CO_2_-independent medium (Invitrogen) with 10% FBS, 2 mM L-glutamine, 50 Units P/S and 10 ng/ml TGF-β1. Imaging was performed at 37°C under normal air conditions using the Nikon TE2000 microscope, and NIS-Elements AR 3.0 software (Nikon) taking images every 10 minutes for 8–10 h.

### Neurite outgrowth on the *in vitro* scar

For the assessment of neurite outgrowth, the scar cultures treated with scar-reducing compounds were co-cultured with cortical neurons and stained with antibodies directed to βIII-Tubulin (Millipore, 1:1000). Using a peroxidase-labelled goat anti-mouse secondary antibody, the signal was detected following incubation with DAB and counterstaining was performed with Haematoxilin. Three to five coverslips per treatment were analyzed. Depending on the thickness of the cluster and the presence of neurites all clusters were photographed at 1–3 different planes either with the Keyence BZ-8000 microscope and corresponding software, or the Olympus BX51 microscope and Stereo Investigator software. For each cell layer, three random pictures were taken per coverslip. Quantification of neurite length was performed using the Neuron J Plugin of Image J. Statistics (one-way Anova with Dunnett’s multiple comparison test) were calculated using the GraphPad Prism 5 software.

### Immunocytochemistry (ICC)

Cell markers, ECM molecules and axon growth-inhibitory proteins were investigated using antibodies directed to GFAP (mouse and rabbit, Millipore and Dako, respectively), fibronectin (mouse and rabbit, Millipore), βIII-Tubulin (Millipore), collagen IV (M3F7, Developmental Studies Hybridoma Bank), BrdU (Cehmicon, for the proliferation assays), collagen I/III/V (F1C3) [41, 42], chondroitin sulphate-56 (CS-56, Sigma), NG-2 (Millipore), neurocan (1F6, a gift from Dr. B. Grimpe), phosphacan (3H1, a gift from Dr. B. Grimpe), semaphorin 3A (sema3A, Abcam), Tenascin C (polyclonal rabbit antibody, batch KAF14) [43]. For permeabilization, 0.2% Triton X-100 (Sigma-Aldrich) was included in the blocking and antibody incubation steps, except for the immune staining of CSPG which tend to be washed off by Triton due to their large sugar chains. Blocking was performed using 1% BSA and 5% of the serum corresponding to the species of origin of the secondary antibody (horse, goat or donkey). Incubation with the primary antibody was performed overnight at 4°C; the secondary Alexa fluorophore-coupled antibodies (Molecular Probes) were incubated for 1 h at room temperature, including DAPI (1:10,000, Roche). Cell markers GFAP, Fibronectin and Tubulin were visualized using Alexa 488- or 594-coupled secondary antibodies. The other antigens were visualized at a wavelength in the infrared spectrum using Alexa 647-coupled secondary antibodies, because the clusters were autofluorescent in the visible light spectrum. Since the CS-56 antibody is an IgM antibody subtype, a biotinylated anti-IgM antibody (Vector Laboratories) and Alexa 647-coupled streptavidin (molecular Probes) were used. Pictures of the stainings were taken using the Keyence BZ-8000 microscope with corresponding software, or the Olympus BX51 microscope with the program MetaMorph.

### Real-time quantitative reverse transcription PCR (qRT-PCR)

RNA was isolated from the co-cultures at 7 days post treatment using the Qiagen RNeasy Mini Kit. After washing the co-cultures with medium the cells were resuspended in 150 μl RLT buffer with β-mercaptoethanol (1:100, Sigma-Aldrich) per well and 3 wells were pooled. Per treatment 7–8 samples were thus produced. Samples were homogenized using 1.4 mm-sized zirconium oxide beads and the Precellys 24 homogenizer (Peqlab). RNA isolation using spin columns was performed according to the manufacturer’s protocol. In-solution DNase treatment was applied immediately after elution for 15 min at 37°C using 20 U of DNase I RNase-free (Roche). The RNA was cleaned using the Qiagen RNeasy MinElute RNA Cleanup kit and 750 ng of RNA was used as template for Superscript III first strand cDNA synthesis (Invitrogen) using a mixture of random hexamer primers (250 ng) and oligo(dT) primers (50 pmol).

Primers were designed using the Beacon Designer software (Premier Biosoft), except for ornithine decarboxylase 1 (ODC1) which was published before [44]. Quantitative PCR reactions were run using the iCycler iQ Thermal Cycler (Bio-Rad) and the corresponding iQ SYBR-Green Supermix as previously described [45]. Briefly, primer concentration, temperature and PCR efficiencies were optimized using a mix of cDNA from all cultures by running temperature gradients and standard curves (Table 1). cDNAs were diluted 4x so that 5 μl of cDNA was added to the reaction mix in a total volume of 25 μl. Reactions were performed in triplicate for each cDNA. The relative levels of the target genes as compared to the reference gene, corrected for the individual efficiencies of the PCRs were calculated using the following equation X0,targetX0,reference=E−Cq,targetE−Cq,reference in which X0 = mRNA levels at cycle 0, E = efficiency, Cq = quantification cycle value [46]. Statistics were calculated using the GraphPad Prism 5 software: one-way Anova with Bonferroni post-hoc test for the treated co-cultures (all treatments compared to the control) and unpaired T-Test for the single cell cultures (TGF compared to no TGF).

**Table 1:** Sequences and optimization data of quantitative PCR primers.

Primer	Sequences	Temp (°C)	Conc (nM)	Efficiency (%)
Collagen IV	CCTCCTGTTGTATTGTTA and GTGTTAGTTACGCAAATC	53	200	95,7
CTGF	GTTACCAATGACAATACCT and TTTGCCCTTCTTAATGTT	58	200	87,1
Cyclophilin	ACGCCGCTGTCTCTTTTC and TGCTGTCTTTGGAACTTTGTC	53	100	86,5
Eph B2	ACCTTCAACCTCTACTACTATG and CTTCACCCACGGATTCTC	58	200	92,9
Ephrin B2	TTCACCATCAAGTTCCAAGA and CCCTCCAAAGACCCATTT	58	200	94,2
GAPDH	GACATCAAGAAGGTGGTGAAG and AGCATCAAAGGTGGAAGAATG	54	200	98,7
Neurocan	CAGACTCCATAGAAATCG and ATTCACTGTCACATTACT	57	100	87,4
NG2	CCAATAGTTCCAGTCTTG and TCATTCCCACTTCGTATA	56	200	90,9
ODC1	GGTTCCAGAGGCCAAACATC and GTTGCCACATTGACCGTGAC	58	200	100,2
Phosphacan	TAATCAAGGAGGAAGACT and TAGTGAGTTGTGGTAGAA	55	400	100,2
Sema3A	AGAGCCTTGGTATATTGG and TTCTGTCCTGATGATATGAT	57	400	93,0
α-SMA	TTATTGCTCCTCCAGAAC and CTTCGTCATACTCCTGTT	57	100	94,0
Tenascin-C	GCAGACATTGATAGTTATAGA and ACTCCATATTCAGTTCCT	57	200	89,6

### Western blotting

Protein isolation was performed by scraping the co-cultured cells into 50 μl of lysis buffer (10 mM Tris-HCl, 10 mM Hepes, 150 mM NaCl, 5 mM EDTA, Complete EDTA-free protease inhibitor cocktail (Roche), 1 μg/ml pepstatin (Sigma), 0.5% NP40 and 1% Triton) per well, pooling 12 wells. Samples were homogenized using 1.4 mm-sized zirconium oxide beads and the Precellys 24 homogenizer (Peqlab), and protein concentrations were measured using Bradford Ultra (Expedeon) and the Infinite M200 Pro plate reader (Tecan). 20 μg of proteins were loaded on the SDS-PAGE gels. We used 4–15% gradient Mini-PROTEAN_TGX precast gels (Bio-Rad) for the detection of α-Tubulin (Neomarkers, Thermo Scientific, 1:2000), collagen (F1C3, 1:1000) and Tnc (KAF14, 1:1000). Semi-dry blotting was performed at 200 mA for 1h and subsequently at 120 mA for 1 h using the Trans-Blot SD Blotter (Bio-Rad). Blocking was performed using 1x Roti-Block (Roth) and primary antibodies were incubated overnight at 4°C. After washing with TBS-T, anti-mouse DyLight-800 and anti-rabbit DyLight-680 secondary antibodies (Cell Signaling) were used at 1:15000 and after washing, the signals were measured using the Odyssey CLx infrared imaging system (LI-COR). Quantification of 3 technical replicates was performed using the Image Studio Lite Western blot analysis software. Statistics: one-way Anova with Bonferroni post-hoc test.

### *In vivo* dorsal spinal cord hemisection and treatment

Dorsal spinal cord hemisections at thoracic level T8 were performed with a Scouten wire knife (Bilaney, Germany) on adult female Wistar rats (200–250 g) as previously described [8, 10] with slight modifications. In brief, under isoflurane anaesthesia (Forene, Abbott, Germany; 2–3% in O_2_ and NO_2_ at a ratio of 1:2) a complete laminectomy of T8, T9 and T11 was performed and the dura mater was opened at T8. The dorsal corticospinal tract and dorsal columns were completely cut to the depth of the central canal. Due to slight asymmetry of the Scouten wire knife, the left rubrospinal tract (RST) is more severely lesioned than the right RST [8, 10]. After suture of the dura mater, an intrathecal catheter was guided in the epidural space from T11, underneath T10, up to the lesion site at T8. 28G polyurethane Alzet rat intrathecal catheters (Charles River Laboratory) were used in studies of collagen IV quantification. In the long-term behavioral study the rats received self-made 32 G polyurethane (ReCathCo) intrathecal catheters. Following catheter fixation and filling with treatments (Table 2), the catheter was inserted into the subarachnoid space in close proximity to the dura suture and connected to a prefilled (Table 2) osmotic minipump (Alzet pump model 1007D, 2001 or 2002) that was placed subcutaneously. Finally, the lesion area was covered with a piece of stretched Nescofilm (Carl Roth) and the overlaying muscle and skin were sutured in layers. Immediately after surgery animals received 5 ml of physiological saline and 5 mg/kg Rimadyl (Pfizer) subcutaneously. Individual caging was provided until the animal had fully recovered from anaesthesia. Post-operative care included manual bladder emptying until normal function returned, prophylactic treatment with antibioticis (Baytril; Bayer Healthcare) for 1 week and pain relief (Rimadyl; Pfizer) for 2 days post-lesion.

**Table 2:** *In vivo* experimental groups.

Analysis	Groups	Alzet osmotic minipump model	Survival time	n
	20 mM TRIS			6
	1.1 μg/d BPY-DCA	2001 (1 μl/h, 7d)	7d	6
	7.8 μg/d BPY-DCA			6
	PBS			6
Coll IV reduction	50 μg/d cAMP	2001 (1 μl/h, 7d)	7d	6
	100 μg/d cAMP			5
	PBS			7
	10 μg/d DFO	2001 (1 μl/h, 7d)	7d	5
	50 μg/d DFO			6
Functional recovery &	PBS	2002 (0.5 μl/h, 14d)	19 w	8*
histological analysis	10 μg/d DFO	2x 1007D (0.5 μl/h, 7d)	19	8*

* Animals showing autotomy are not listed

### Anterograde CST axon tracing

At the end of behavioral testings (16 weeks post-lesion), corticospinal tract (CST) labeling was performed by multiple microinjections (0.2 μl each for 2 min) of biotinylated dextran amine (BDA; 10 000 MW; 10%; Molecular Probes) into the sensorimotor cortex layer V. Eight injections were made stereotactically (Small Animal Stereotaxic Frame, Kopf Instruments) into each hemisphere using the coordinates previously described [8]. After three weeks (19 weeks post-lesion) the traced animals were sacrificed.

### Immunohistochemistry (IHC)

Deeply anaesthetized rats were transcardially perfused with ice-cold 0.1 M PBS for 2 min followed by 4% paraformaldehyde (PFA, Merck) for 12 min. Spinal cord pieces of ~ 2 cm length including the lesion area were collected and post-fixed for 24 h at 4°C. Afterwards, the spinal cord tissue was either embedded in paraffin (Merck) for histological investigation of the lesion scar 7 days post-lesion or in 10% gelatin (BD) in 0.1 M PB with 0.1% sodium azide (Merck) for evaluation of axon regeneration and tissue sparing 19 weeks post-lesion.

#### Spinal cord tissue 7 days post-lesion

Paraffin-embedded spinal cord tissue was cut into serial 10 μm parasagittal sections with a paraffin-microtome and subsequently double-stained for collagen IV (M3F7, Developmental Studies Hybridoma Bank, 1:500) and von Willebrand factor (vWF, Dako, 1:500). The immunohistochemical staining of paraffin sections was started by deparaffinization procedures followed by a standard immunohistological staining protocol. Briefly, after washing with PBS and antigen retrieval with 0.05% protease XXIV for 8 min at 37°C, sections were blocked with 5% donkey serum for 1 h at room temperature. Primary antibodies were incubated overnight at 4°C and after washing with PBS, incubation with Alexa 488 and 594-conjugated secondary antibodies (Molecular Probes, 1:500, respectively) was performed for 1 h at room temperature. For reduction of autofluorescent background, sections were additionally stained with 0.3% Sudan Black dye (Fluka).

#### Spinal cord tissue 19 weeks post-lesion

Gelatin-embedded spinal cord tissue was parasagittally cut into serial 50 μm thick free-floating sections using a vibratome. After blocking with 5% donkey serum for 1 h at room temperature, BDA-traced axons were visualized by Oregon Green 488 dye incubation (Molecular Probes, 1:1000), ascending axons were stained by anti-CGRP (calcitonin gene related peptide) (AbD Serotec, 1:1500) and every section was additionally stained for GFAP (Chemicon, 1:500) overnight at 4°C to identify the GFAP-negative lesion area. For infrared visualization of GFAP, an Alexa 647-conjugated secondary antibody (Molecular Probes, 1:500) was used and incubated for 2 h at room temperature. All antibodies were diluted in PBS containing 0.3% Triton X-100 and 5% donkey serum.

### Collagen IV quantification in the scar

For histological quantification of lesion-induced collagen IV (Coll IV) deposition in the scar, the complete scar area of parasagittal spinal cord sections, double-stained for Coll IV and vWF, was photographed at 10x magnification using the mosaic scan function of the BZ-8000 Keyence microscope. All images were taken with the same exposure times and were converted into 16-bit greyscale pictures. In order to quantify the amount of Coll IV in the extracellular matrix, excluding Coll IV in the blood vessel endothelium, we subtracted the vWF positive blood vessels from the Coll IV-stained area, using Image J Software. The vWF pixels, highlighted by an individually adapted threshold, were subtracted from the corresponding greyscale Coll IV image. After framing the scar area, the Coll IV area fraction was measured at a constant threshold value, which was determined by averaging individually identified threshold values in sections of control animals (no treatment). A total of six spinal cord sections per animal were analyzed. Statistics were calculated using the GraphPad Prism 5 software: one-way Anova with Dunnett’s post-hoc test

### Quantification of regenerating axons

Every 6^th^ parasagittal spinal cord section (4–6 sections per animal) of BDA-traced animals as well as sections stained for CGRP by IHC was used to quantify axon growth within the GFAP-negative lesion area. Axon profiles were manually counted throughout all focal planes using the BZ-8000 Keyence microscope. Only positively stained structures which could clearly be identified as axons due to specific regenerative characteristics [47] were considered for evaluation. The efficiency of the BDA tracing of the CST was checked by quantification of the number of stained CST axon profiles in 20 μm coronal sections of the rostral spinal cords of 3 randomly chosen animals per treatment group (5 coronal sections per animal). For this purpose, we images of BDA-traced axons, visualized by Oregon Green 488 dye incubation (Molecular Probes, 1:1000), were photographed using the same exposure time and post-processed in ImageJ using the “image of interest background correction” tool. The number of BDA-stained axon particles (cross-sections) in the dorsal CST area was counted by automatic particle counting tool of Image J software and divided through the analyzed area (mm^2^). Similar to the collagen IV quantification described above, a threshold was set using ImageJ software, and the number of positively stained pixels per mm^2^ was measured. Statistics were calculated using the GraphPad Prism 5 software: unpaired T-test

### Assessment of lesion area and spared tissue

For evaluation of lesion and spared tissue areas, GFAP-stained parasagittal spinal cord sections (50 μm) were used, as the fibrous scar is characterized by a lack of GFAP expressing astrocytes [8]. The lesion and spared tissue areas were determined according to methods described before [48] and [40] with slight modifications. From each spinal cord, the section containing the transected central canal and two additional sections in 0.2 mm distance left and right to the central canal were taken for quantification. The complete lesion area of the spinal cord was captured at 5x magnification using the mosaic scan function of the BZ-8000 Keyence microscope. In all merged images, the total area of spinal cord, which was limited to a total length of 2.5 mm in rostral-caudal direction, was measured with ImageJ software and used as reference area. To determine the percent lesion area, lesion area (mm^2^) was divided by the reference spinal cord area (mm^2^) from the same section. Spared tissue area (mm^2^) was calculated by subtracting the area covered by the lesion from the reference spinal cord area. Statistics were calculated using the GraphPad Prism 5 software: unpaired T-test

### Behavioral testing

Four weeks prior to surgery adult female Wistar rats (n = 24) were familiarized and pre-trained in three different behavioral tests: (1) the Basso-Beattie-Bresnahan (BBB) open field test, (2) the horizontal ladder (gridwalk), and (3) the CatWalk gait analysis. Three animals developed severe autotomy during functional testings and were killed prematurely. Three animals had to be excluded due to unstable catheter fixation and two animals died during/after surgery. Because of the Scouten wire knife-induced lesion asymmetry [10], where the right RST is less injured than the left RST, the hindlimbs were evaluated separately. All behavioral tests have been performed and analyzed blinded to the treatment groups. Statistics were calculated using the GraphPad Prism 5 software: Mann-Whitney Test after D’Agostino and Pearson omnibus normality test (not passed, therefore a non-parametric test was required)

#### BBB open field test

The overall hindlimb function was assessed in an open field using the Basso-Beattie-Bresnahan (BBB) score [49]. Freely exploring rats were observed by two examiners blinded to the treatment at 1, 2, 12 and 16 weeks post-lesion. Due to the difficulty of the correct assessment of coordination during BBB observation, forelimb-hindlimb coordination was determined separately using the regularity index (RI) measured in the CatWalk test. According to [50], the RI-based coordination was defined as occasional, when the animal had performed at least one out of four CatWalk runs with 100% RI; as frequent, when an average of 95% RI or above was reached and two out of four runs being at 100% RI; and for consistent coordination the animal had to have an average RI of 95% or more and at least 3 runs being at 100% RI. As we expected deficits in normal over ground locomotion primarily in fine motor control aspects due to our lesion type, we also assessed the 7-point BBB subscore [51]. Normal over ground locomotion is mainly controlled by the ventrolateral localized central pattern generator and its reticulospinal input [52, 53], which are not or only insignificantly affected by our lesion model.

#### Horizontal ladder walking test

The horizontal ladder walking test was performed according to established methods [54, 55]. To prevent animals from learning a movement pattern, the irregular bar distances were changed in every testing week. The walking performance of each animal was recorded with a conventional video camera and subsequently analyzed in slow motion by counting the number of missteps (errors) of each hindlimb in relation to the total number of steps. A misstep was counted when the paw slipped or fell off the bar. If an animal was not able to cross the ladder, a maximum error rate of 100% was given. Both hindlimbs were evaluated separately in four uninterrupted crossings per animal and per time point. Baseline data were collected in the last week prior to surgery and the postoperative testing period started two weeks post-lesion with a two-week testing rhythm.

#### CatWalk gait analysis

Differences in walking patterns were studied according to Hamers et al. (2001) by using the CatWalk device and software from Noldus Information Technology [56]. Animals were trained to cross without interruption a horizontal glass walkway, which is equipped with the Illuminated Footprints technology. Illuminated paw prints were captured by a high speed color camera underneath the walkway. Recordings of the animals`gait were taken in the last week prior to surgery (baseline) and every second week post-lesion as described for the horizontal ladder test. In four uninterrupted runs per animal and per time point differences in walking patterns were assessed with the regularity index (expressed in percentages). The regularity index (RI) is a measurement of interlimb coordination, as it is calculated from the number of normal step sequence patterns in relation to the total number of paw placements. An RI of 100% represents perfect coordination [56].

## Results

### Characteristics of the in vitro scarring model

For the present investigation, we modified the meningeal fibroblast-cortical astrocyte co-culture model of Kimuro-Kuroda et al. (2010) [19]. We changed the cell culture substrate from the plastic Lab-Tek chamber slides to less autofluorescent glass coverslips, which provided improved attachment of the fibroblasts. Additionally, we reduced the number of plated cells (15,000 instead of 50,000 each) to avoid detachment of the cell droplets after addition of medium. As in the original paper, the cells needed 7–14 days (10 d on average) to form monolayers that contacted each other. Clusters started to form within 24 h after stimulation with 10 ng/ml TGF-1 and gradually increased in number during 7 days (Fig 1), whereas without the addition of TGF no clusters were formed (Fig 1A–1D). The clusters that formed at the border of the two cell layers consisted of fibroblasts that were surrounded by astrocytes (Fig 1E–1H). Notably, clusters also appeared in the fibroblast layer, away from the astrocytes, where they exclusively consisted of meningeal fibroblasts. The TGF-β inhibitor LY364947 completely abolished cluster formation (Fig 1I–1L), indicating that consistent with the original model the cluster formation is TGF-β-dependent. We studied the number of clusters and their composition at 7 days after stimulation with TGF. In addition, earlier time points (6–24 h) were performed for live imaging and proliferation assays. The effects of anti-scarring treatments were investigated 7 days after simultaneous application of TGF and respective treatments. To study neurite outgrowth on the scar-like clusters, dissociated neonatal cortical neurons were plated onto the co-cultures at 7 days after TGF stimulation and scar-reducing treatments and were then allowed to grow for another 3 days.

### Scar-like clusters are formed by proliferation and reorganization of the fibroblast layer

**Fig 2:**
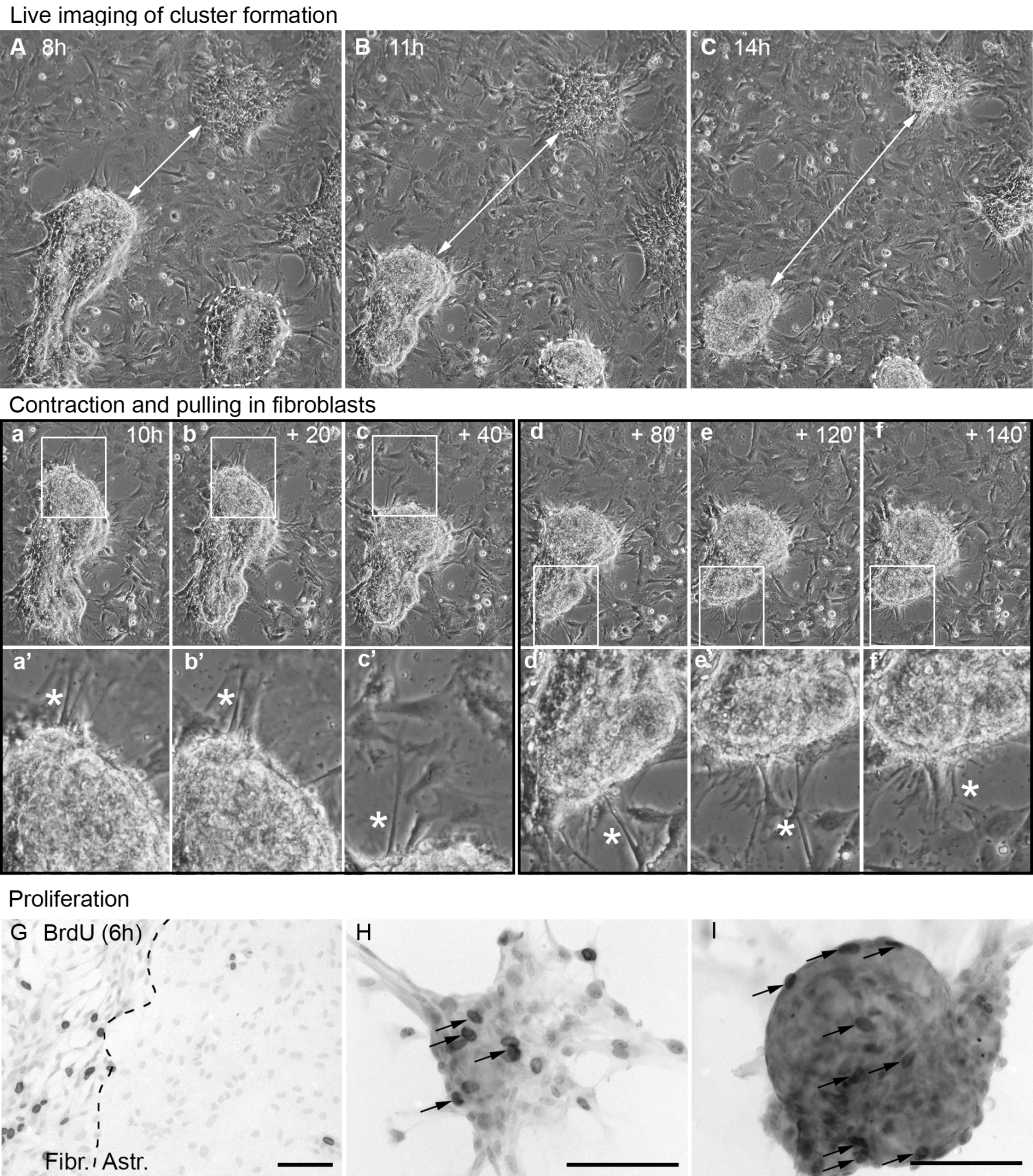
Mechanisms of cluster formation. Selected frames from live imaging of the co-cultures from 8 h to 14 h after TGF-β stimulation (S1 movie). (A-C) Accumulations of meningeal fibroblasts developed into clusters that contracted over time. White arrows indicate the distance between two clusters, illustrating their contraction. Dashed line: cluster contracting to about half the size. Waves of cluster contractions are observed, for example from 10 h (a) to 10 h + 20 min and 10 h (b) + 40 min (c) and from 10 h + 80 min (d) to 10h + 120 min (e) and 10 h + 140 min (f). During a contraction phase adjacent fibroblasts (asterisks) first elongated and then integrated into the cluster (a’-f’, corresponding to boxed areas in a-f). (G-I) Proliferation was visualized by labeling with BrdU for 6 h (dark nuclei). Cultures were counterstained with haematoxilin (faint nuclei). (G) No TGF: meningeal fibroblasts (Fibr.) proliferate more than cortical astrocytes (Astr.). (H) TGF: BrdU-labeled nuclei (arrows) in an accumulation of fibroblasts (cluster precursor) and (I) TGF-induced cluster showing many BrdU-labeled fibroblasts (black arrows). Scale bar = 100 μm.

Since we observed that large cell-free gaps appeared around the clusters during their formation, we investigated fibroblast migration behavior using live cell imaging. For this purpose we cultured the cell layers for 10–14 days under CO_2_-conditions, but then changed to CO_2_-independent medium at the point of TGF-β addition, since the imaging was performed in a chamber with normal air. From 6 hours after TGF addition onward, microscopic images were automatically taken every 10 minutes during the following 8 hours. Interestingly, we did not observe clear-cut migration of fibroblasts into the cluster. Rather, a striking reorganization of the fibroblast layer was observed. While in some cases sheaths of fibroblasts seemed to partially detach and form 3-dimentional structures, in other cases fibroblasts grew on top of each other. The clusters thus formed often contracted to about half their size (Fig 2A–2C, S1 movie). This contraction occurred in waves, as illustrated in Fig 2A–2F where specific frames from 10 h + 20 min to 10h + 40 min and from 10h + 80 min to 10 h + 140 min are shown. During these contraction phases attached neighboring fibroblasts were pulled into the clusters (Fig 2A’–2F’). The contraction led to clearly demarcated condensed clusters. There was no obvious active movement of cells from the surrounding cell layer towards the clusters, suggesting that reorganization and contraction were the main mechanisms of scar-like cluster formation.

At 24 h after stimulation with TGF-β the 8-hour imaging period showed the further reorganization of pre-formed clusters. The latter were still contracting and compacting and those clusters that were situated near the astrocyte-fibroblast border were being pulled towards the astrocytes (right field in S2 movie), suggesting an active participation of the astrocytes near the border in cluster formation. Again, it was observed that fibroblasts that were attached to the cluster first held on to the cell monolayer prior to elongating and finally being pulled into the cluster (S2 movie).

In order to further investigate the mechanisms of scar formation, we incubated the co-cultures with BrdU for 6 hours. Without the addition of TGF-β fibroblasts proliferated much more actively than astrocytes (Fig 2G). After simultaneous incubation with TGF-β and BrdU, we found that cluster precursors (defined as a non-condensed accumulations of fibroblasts, Fig 2H) as well as mature clusters (condensed accumulations of fibroblasts, Fig 2I) contained numerous BrdU–positive cells, indicating that the clusters are comprised of proliferating fibroblasts.

### Scar-like clusters contain ECM molecules and axon growth inhibitors

**Fig 3:**
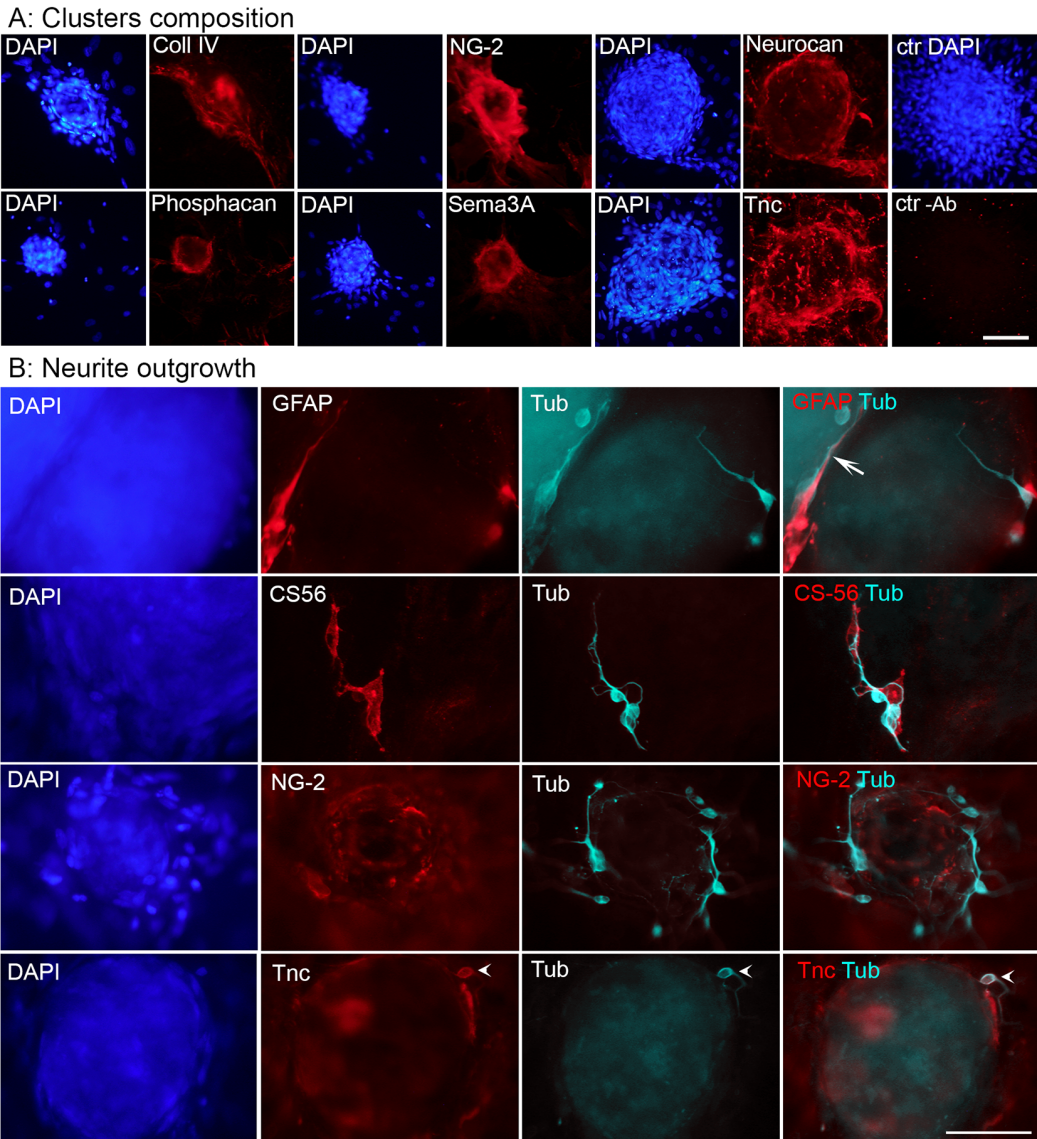
Cluster composition, correlation with neurite growth. (A) ICC for selected ECM and axon growth inhibitory molecules stained with infrared-labelled secondary antibodies in the 7-days-old clusters. Scale bar = 100 μm. (B) Co-culture with neonatal cortical neurons. ICC staining with GFAP, CS-56 (a general marker for CSPGs), NG-2 and Tnc in infrared, β-III-Tubulin in cyan, DAPI in blue. Neurites tended to co-localize with CSPG-positive astrocytes (arrow). Neurites were short and avoided the cluster center. Tnc was expressed by the neurons (arrowheads) as well as by the fibroblasts. Scale bar = 50 μm.

To confirm that the composition of the clusters resembled the lesion scar in SCI, we performed immunocytochemistry (ICC) for several ECM molecules and axon growth inhibitors known to accumulate in the fibrous scar *in vivo*. Due to high autofluorescence in the green wavelength and some autofluorescence in visible red, all scar components were visualized with infrared dyes (Alexa 647). We observed immunoreactivity for Coll IV, Tnc, and Sem3A as well as for the CSPGs NG-2, neurocan and phosphacan (Fig 3A and 3B). Ephrin B2 and EphB2 were expressed at very low levels (data not shown) and controls lacking primary antibodies were negative (Fig 3A last panels). For most molecules, higher levels of immunoreactivity were observed at the border and surface of the clusters compared to the center (Fig 3A). Dissociated neonatal cortical neurons grew βIII-Tubulin-positive neurites on both fibroblast and astroglial cell layers. Neurite growth was less pronounced on top of the clusters and often confined to areas where astrocytes were present (Fig 3B). Neurites mostly did not cross the border between the monolayer and the cluster, where often high concentrations of growth inhibitors were found (Fig 3A and 3B).

### TGF-β induces mRNA expression of scar components

**Fig 4:**
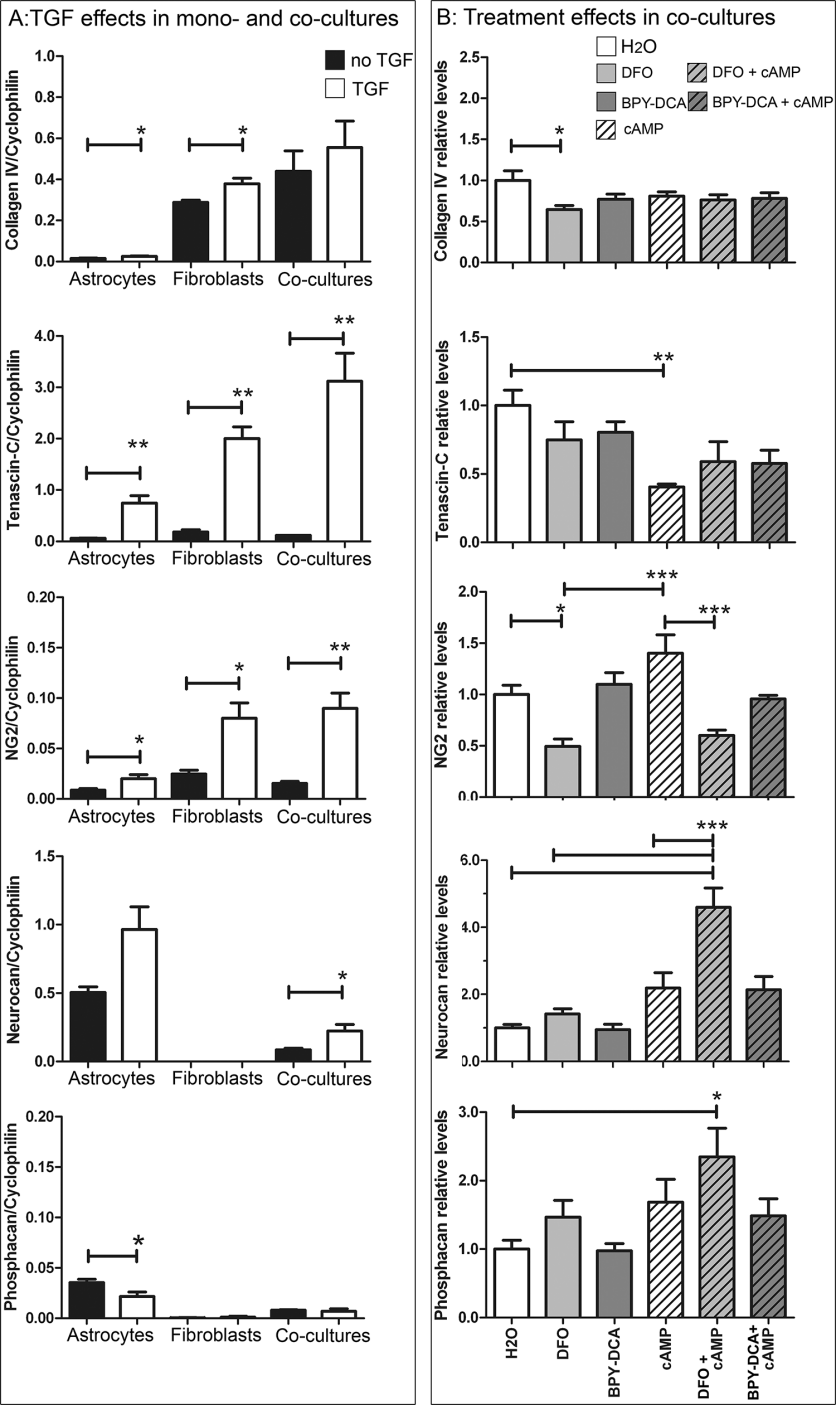
Effects of TGF-β and scar-reducing treatments on gene expression. (A) Effects of TGF-β on the mRNA expression of ECM molecules and axon growth inhibitors in cortical astrocytes and meningeal fibroblasts cultured separately and in the co-cultures. Levels of each target mRNA were normalized to the housekeeping gene *cyclophilin*. Statistics: unpaired T-test * p < 0.05, ** p < 0.01. (B) Effects of scar-reducing treatments. Plotted are the levels of target mRNAs normalized to *cyclophilin* relative to the H_2_O control treatment. DFO reduced the levels of *collagen IV* and *NG-2*. Treatment with cAMP led to a reduction in *Tnc*. *Neurocan* and *phosphacan* were upregulated in DFO + cAMP, whereas *NG-2* was significantly reduced after DFO treatment and in DFO + cAMP co-treatments compared to cAMP alone. Statistics: one way Anova with Bonferroni * p < 0.05, ** p < 0.01, *** p< 0.001.

We studied the mRNA expression of various ECM molecules and axon growth inhibitors by real time quantitative PCR (Fig 4, Panel A). We extracted RNA from the astrocyte-fibroblast co-cultures as well as from the individual cell mono-cultures, treated with and without TGF-β, in order to investigate the relative contribution of the two cell types. We normalized to *cyclophilin*, a housekeeping gene that stayed constant after TGF treatment as opposed to *GAPDH* and *ODC* which were both regulated by TGF (data not shown). The relative expression levels of the molecules of interest allowed us to estimate their relative contribution to the scar matrix.

We first looked at *coll IV* expression (Fig 4, panel A). Fibroblasts expressed about 18-fold more *coll IV* mRNA than astrocytes, which was slightly upregulated after addition of TGF. In the co-cultures, there was no significant upregulation of *coll IV* mRNA upon TGF addition. *Tnc* mRNA expression was higher than that of *coll IV*. *Tnc* was expressed in both cell types (3-fold higher in fibroblasts) and about 10-fold upregulated upon TGF-β stimulation in the single cell types as well as in the co-cultures. The CSPG *NG-2*, was expressed at intermediate levels by both cell types and significantly upregulated in both cell types after addition of TGF-β (Fig 4, panel A). NG-2 was upregulated 4.4-fold in the co-cultures after TGF stimulation. *Neurocan* was expressed in astrocytes at similar levels as *coll IV*, but could not be detected in fibroblasts. TGF-β induced a 2.3-fold increase of *neurocan (NC)* mRNA in the co-cultures. Although the TGF-β-induced increase in the astrocyte monolayer was not significantly different, the induction in the co-cultures is most likely attributable to the astrocytes. The third CSPG of interest, *phosphacan (PC)*, was expressed in astrocytes at levels about 10-fold lower than those of *neurocan*. Fibroblasts also expressed *phosphacan*, but at 100-fold lower levels than astrocytes. TGF-β addition seemed to slightly decrease the levels of *phosphacan* in astrocytes, but not in the co-cultures.

*Eph B2* and *Ephrin B2*, as well *semaphorin 3A* (*sema3A*) were expressed at the lowest levels, consistent with the ICC results. *Eph B2* and *Sema3A* were slightly downregulated (60 and 77% respectively), whereas *Ephrin B2* was upregulated 2-fold in the co-cultures only but not in the single cell types, where TGF did not induce any change (S1 Fig). This suggests an interaction of the two co-cultured cell types regulating the expression of some of the studied genes.

Altogether, TGF-β upregulated most of the ECM molecules and axon growth inhibitors investigated, but also downregulated some. Both astrocytes and fibroblasts contributed to the expression of most molecules, except for *neurocan* and *phosphaca*n, which were almost exclusively expressed in astrocytes. In some cases (sema3A, Eph B2), the presence of both cell types in the co-cultures clearly led to differential regulation of ECM mRNAs in comparison to the single cell types, suggesting a decisive role of astroglial-fibroblast interactions underlying the significant TGF-β induced gene regulation.

### DFO and cAMP lead to differential expression of axon growth-inhibitory molecules

We tested various putative scar-reducing treatments that were added to the fibroblast-astrocyte co-cultures simultaneously with TGF-β and incubated for 7 days. Treatments consisted of the iron chelators DFO and BPY-DCA alone, cyclic AMP (cAMP) alone, or combinations of these iron chelators with cAMP. The scar-reducing treatments were compared to TGF + H_2_O, the vehicle for most treatments. The effects of the treatments on the modulation of mRNA expression of ECM components and axon growth inhibitors were studied at 7 d post treatment (Fig 4, panel B) by qRT-PCR. DFO significantly reduced *coll IV* mRNA by 46%. The other treatments only showed a non-significant downward trend of *Coll IV* expression. *Tnc* was significantly reduced by 43–60% after cAMP treatment. No additional effects of iron chelators on *Tnc* expression could be observed. In contrast, the mRNA levels of *NG-2* were reduced by DFO and combinations thereof (43–50%), whereas cAMP had no effect on NG-2 expression. *Ephrin B2*, *Eph B2* and *sema3A* expression levels were not changed by any of the treatments (S1 Fig). Finally, both *neurocan* and *phosphacan* were significantly upregulated by the combination of DFO + cAMP.

Using this *in vitro* model for fibrotic scarring it could be demonstrated that the various individual or combinatorial treatments had differential effects on the mRNA expression of ECM and axon growth inhibitory molecules. Among the iron chelators only DFO showed significant effects on mRNA expression of both the ECM molecules and axon growth inhibitors.

### DFO and cAMP reduce cluster formation

The above-mentioned putative scar-reducing treatments were then analyzed with respect to scar reduction. Initially, treatments with TGF alone and TGF + Tris buffer were also included, since the vehicle for BPY-DCA was Tris buffer. TGF alone or in combination with Tris buffer or with H_2_O all led to an average of 40 clusters. Therefore, all following experiments were conducted with TGF + H_2_O controls. A pilot study (data not shown) using 10, 20, 50 and 100 μM of DFO revealed that this iron chelator reduced cluster formation already at 10 μM. Concentrations higher than 50 μM led to detachment of the cells from the plate. BPY-DCA did not reduce cluster formation at all concentrations, but since 100 μM BPY-DCA caused the cells to detach, 50 μM was used in the following experiments. cAMP reduced the number of clusters at 1 and 0.5 mM, but not at 0.25 and 0.1 mM. Since 1 mM induced strong differentiation of the astrocytes, forming extremely elongated parallel fibers (data not shown) we continued the study with 0.5 mM of cAMP. For the experiments presented here, we used 10 μM DFO, 50 μM BPY-DCA and 0.5 mM cAMP as well as combinations thereof. Since all treated co-cultures contained TGF-β1, we refer from this point onward to the treatments only (H_2_O, DFO, BPY-DCA, cAMP and combinations).

**Fig 5:**
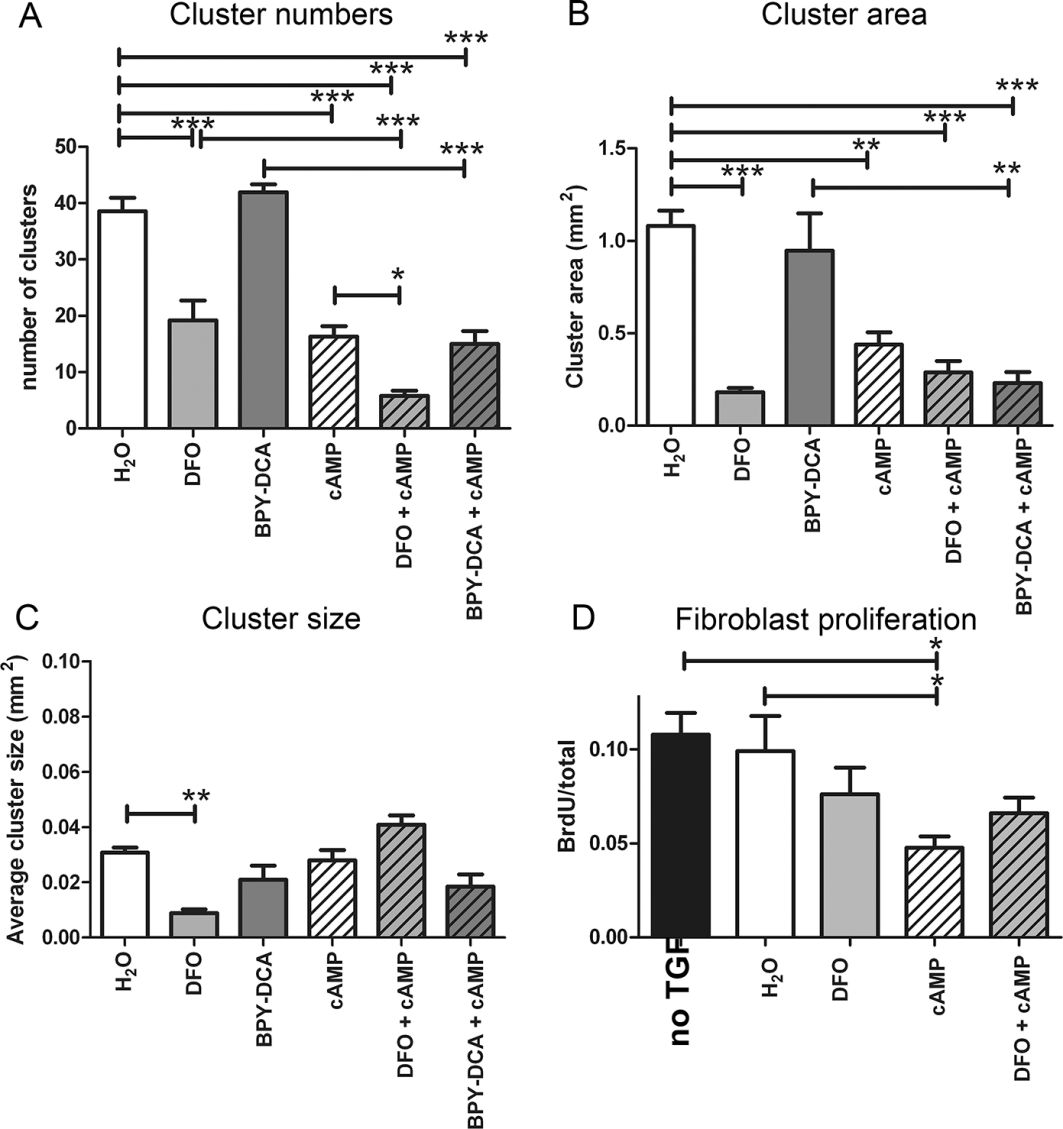
Quantification of scar-reduction. Quantification of (A) the number of clusters, (B) the cluster area and (C) the cluster size after 7 days of TGF-β1 and potential scar-reducing treatments. DFO reduced cluster size, number and area. cAMP reduced the number of clusters but not their size. DFO + cAMP reduced the number of clusters significantly more than DFO or cAMP alone. (D) Effects of scar-reducing treatments on fibroblast proliferation. cAMP reduced the proliferation of the fibroblasts. Statistics: one way Anova with Bonferroni post-hoc test * p < 0.05, ** p < 0.01, *** p< 0.001.

Co- cultures treated with the H_2_O vehicle control contained on average 38 clusters of about 0.03 mm^2^ in size (Fig 5). Treatment of the co-cultures with DFO led to a reduction in the number of clusters by 50% (Fig 5A). The effects of the DFO treatment were quite variable, since in some cases there were around 10 regular-sized clusters and in other cases there were 40 very small clusters. Therefore, we also measured the total area the clusters occupied and the average cluster size using Image J software. Both the cluster size and area were significantly reduced for DFO (71% and 83% respectively, Fig 5B and 5C). BPY-DCA alone affected neither cluster number nor size. cAMP led to a 59% reduction in cluster area which was further reduced (79%) in co-cultures treated with DFO + cAMP. This combination was also significantly different from the single treatments, indicating that DFO and cAMP had additional effects. The combination of BPY-DCA + cAMP showed a significant reduction in cluster number and cluster area compared to the H_2_O control but had no additional effect compared to cAMP alone.

As one of the possible mechanisms of this observed reduction in scar-like clusters, we studied the effects of the treatments on astroglial and fibroblast proliferation in the co-cultures (Fig 5D). For that purpose, we incubated the co-cultures for the first 6 hours after TGF-β addition with BrdU, counterstained with haematoxilin and calculated the proportion of BrdU-labelled nuclei in 3 different areas of each cell layer on 3 coverslips. We found no change in BrdU-labelling in astrocytes (data not shown), whereas a significant reduction of fibroblast proliferation was observed with cAMP, but not with DFO (Fig 5D).

### DFO and cAMP change protein levels of ECM molecules

**Fig 6:**
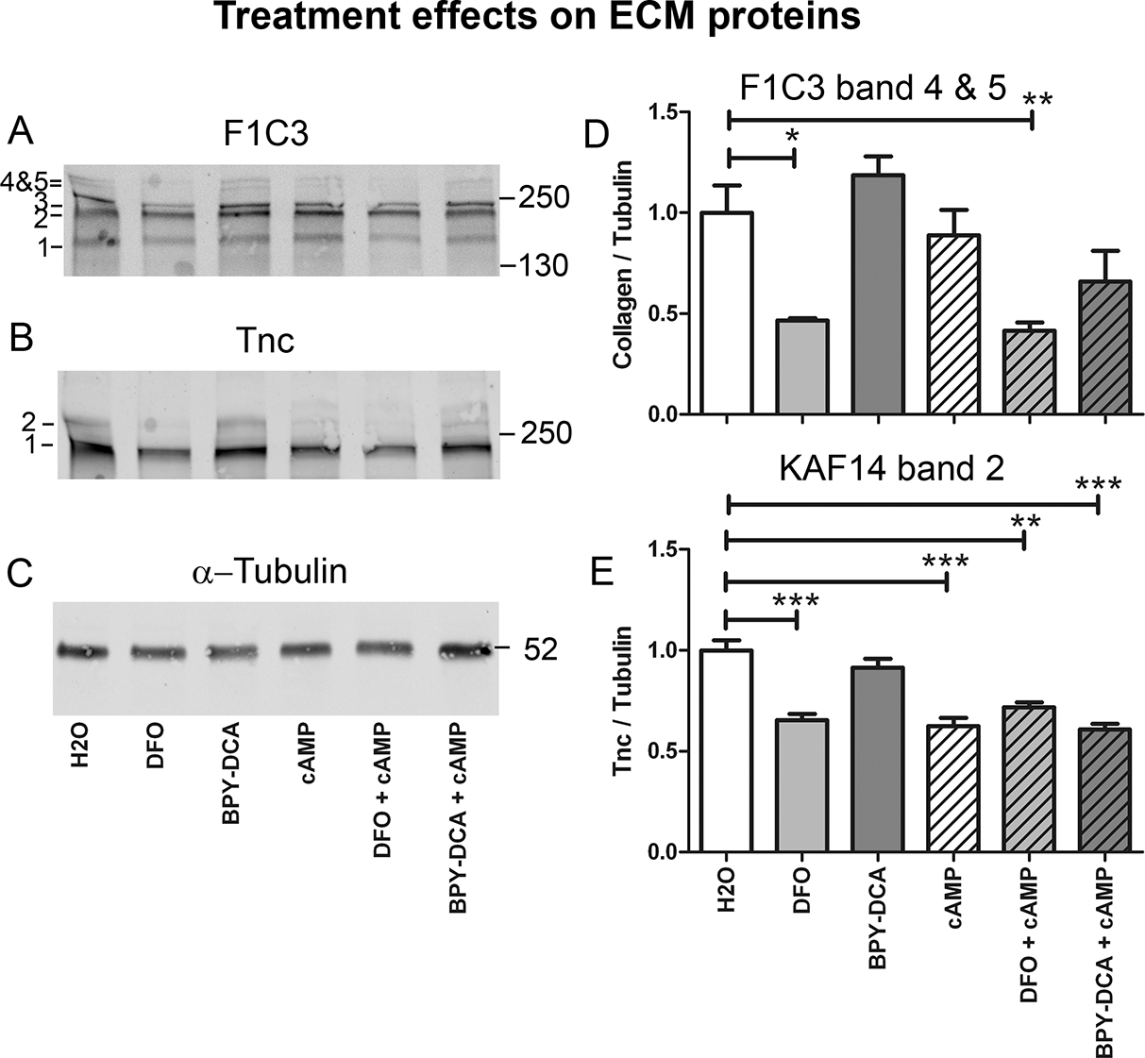
Effects of scar-reducing treatments on protein expression. Effects of treatments on protein levels of ECM molecules. (A) Collagen I/III/V was detected by F1C3 antibody, which stained 5 bands corresponding to collagen polypeptide chains that contribute to the three collagen subtypes. (B) Tnc was detected with KAF14 antibody which stained two bands. (C) α-Tubulin served as a loading control. (D) The upper two collagen bands were reduced by DFO. The reduction in DFO + cAMP was exclusively due to DFO, since cAMP alone did not change collagen levels. (E) The upper band of Tnc was reduced by DFO, cAMP and combinations thereof. Statistics: one way Anova with Bonferroni post-hoc test * p < 0.05, ** p < 0.01, *** p< 0.001.

The influence of the scar-suppressing treatments on the protein expression of the ECM molecules collagen and Tnc was studied in more detail using the F1C3 and KAF14 antibodies, respectively. The F1C3 antibody detected five bands in total of estimated 180, 210, 230 and >250 kDa in size, representing the various collagen polypeptide chains that are present in collagen I, III, and V [42]. DFO, alone or combined with cAMP, significantly reduced the levels of the upper two collagen bands (Fig 6A and 6D). Two Tnc protein bands were detected by KAF14, of estimated 230 and >250 kDa in size. The upper band was significantly reduced by DFO, cAMP and combinations thereof. (Fig 6B and 6E).

### Enhanced neurite outgrowth on the clusters following DFO treatment

**Fig 7:**
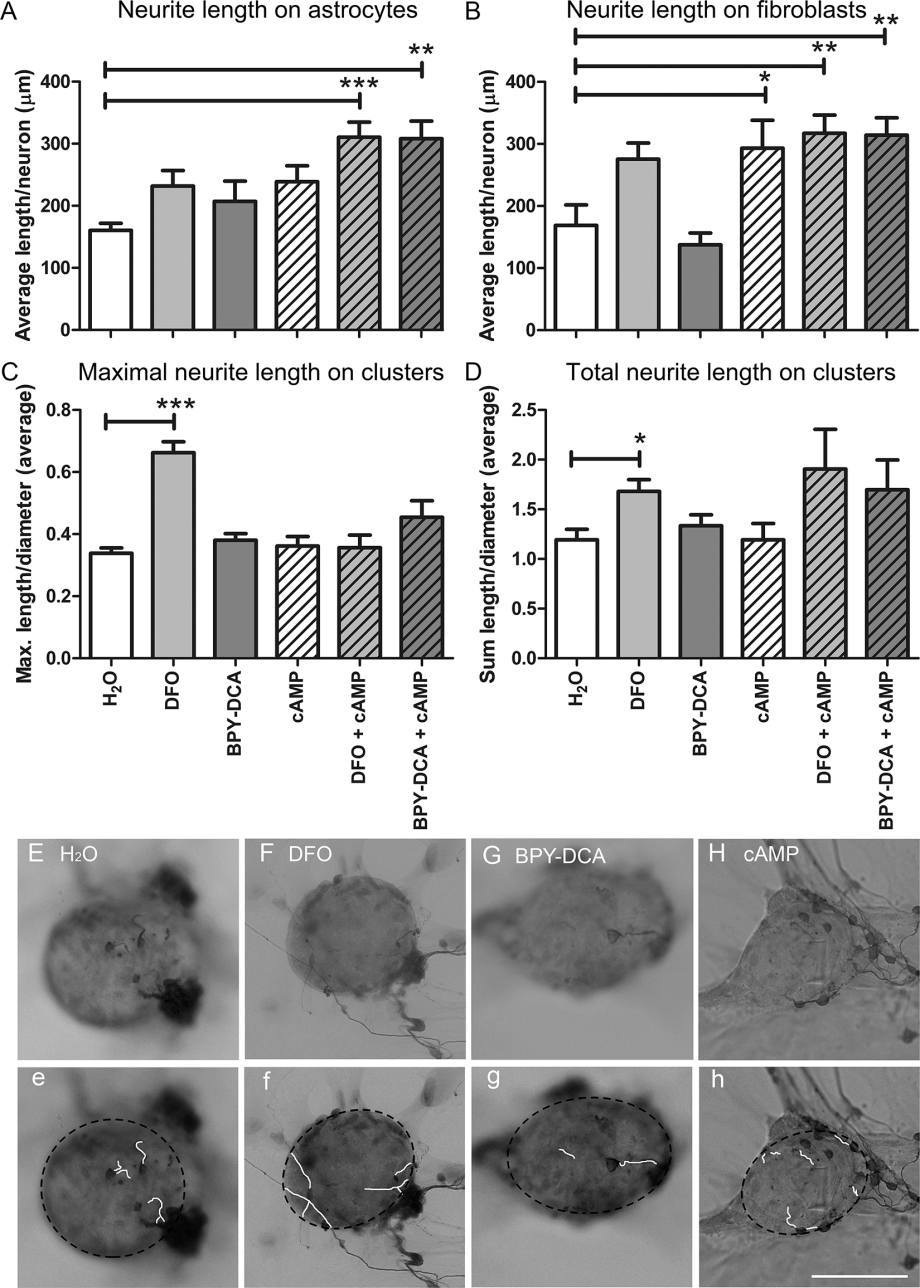
Effects of treatments on neurite outgrowth. Neurite outgrowth from neonatal cortical neurons on the fibroblast and astrocyte cell layers as well as on the clusters in the co-cultures after treatment. cAMP and the combination treatments had a positive effect on outgrowth on the fibroblast layer, whereas only the combination treatments resulted in increased growth on the astrocytes (A, B). DFO was the only treatment resulting in increased growth of neurites on the clusters. Corrected for the cluster diameter, DFO showed an increase in the maximal length (C) and the sum length of the neurites (D). Representative pictures of clusters with neurites (E-H) that were traced using the Neuron J plugin of Image J (e-h, clusters marked by dotted black lines). Statistics: one way Anova with Dunnett’s post-hoc test * p < 0.05, ** p < 0.01, *** p< 0.001 Scale bar = 100 μm.

In order to study putative axon growth-permitting properties of the scar-reducing treatments, we quantified neurite outgrowth of neonatal cortical neurons plated onto the co-cultures (Fig 7). On the astrocyte layer, only the combination of iron chelators with cAMP significantly increased the average neurite length per neuron. On the fibroblast layer, cAMP alone or in combination with iron chelators increased neurite length significantly. Only a non-significant trend towards longer neurites on fibroblasts was observed upon DFO-treatment.

We then measured the length of neurite segments growing on the scar-like clusters. The overall length of the neurites had to be normalized to the cluster diameter, since axons crossing smaller clusters would per definition be scored shorter. The co-cultures treated with DFO alone displayed the longest neurites in relation to the cluster size (maximal length/diameter, Fig 7). Additionally, the total length of neurite segments on the clusters was significantly increased upon DFO treatment (sum length/diameter, Fig 7). None of the other treatments resulted in significant changes in neurite growth on the clusters, although a non-significant trend was observed for DFO + cAMP.

### Scar reduction *in vivo*

**Fig 8:**
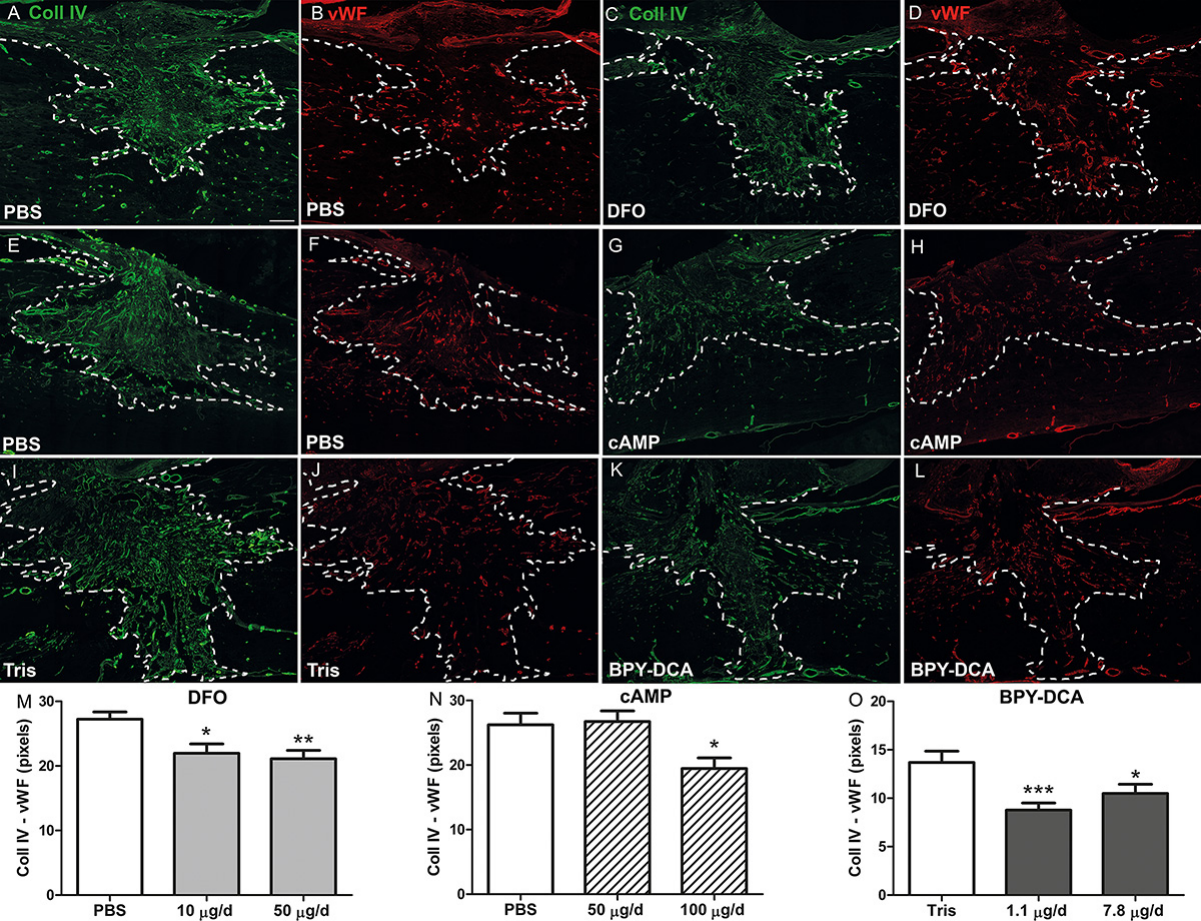
Reduction of scar density measured by the quantification of ECM Coll IV. Mosaic composite pictures of the complete scar in spinal cord injured animals after treatment with (A, B) PBS corresponding to (C, D) DFO treatment, (E, F) PBS corresponding to (G, H) cAMP treatment and (I, J) Tris corresponding to (K, L) BPY-DCA treatment. The tissue was stained for Coll IV in green (A, C, E, G, I, K) and vWF in red (B, D, F, H, J, L). Quantification of ECM Coll IV was performed by subtraction of vWF-positive blood vessels. The remaining signals for ECM Coll IV for the respective treatments were plotted in M, N and O. A significant reduction of Coll IV was observed in both treatment concentrations for BPY-DCA and DFO as well as in the highest concentration of cAMP. High magnification pictures of the Coll IV staining in part of the lesion site are provided in S2 Fig. Statistics: one way Anova with Dunnett’s post-hoc test * p < 0.05, ** p < 0.01, *** p< 0.001 Scale bar (in A) = 200 μm, applies for all pictures.

The *in vitro* results suggest that DFO treatment may be preferable over the existing AST strategy with respect to reduction in number and size of scar-like clusters as well as neurite length on clusters. The combination of iron chelators with cAMP reduced the number of clusters slightly more, but also led to undesirable upregulation of *phosphacan* and *neurocan* mRNA. To prove the efficacy of DFO *in vivo* we investigated the scar-reducing capabilities of DFO in comparison to BPY-DCA and cAMP in the dorsal hemisection spinal cord injury model in rats. The treatments were applied by local intrathecal infusion for 1 week using osmotic minipumps. We applied 10 and 50 μg DFO per day, which would correspond to roughly 30 and 150 μM assuming a CSF volume of 500 μl in rat [57]. For BPY-DCA, the 1.1 or 7.8 μg/d would be about 9 or 70 μM. The amount of cAMP applied was 50 and 100 μg/d (0.2 and 0.4 mM). These calculations fall roughly within the range of the concentrations used in the *in vitro* model. As a measure for scar density we then semi-quantitatively determined the amount of Coll IV in the ECM of the scar by immunohistochemistry [8]. For this purpose, we double stained with an antibody directed to vWF to correct for the amount of blood vessels in the scar (Fig 8). Since Coll IV is also expressed in endothelial cells [58, 59] and iron chelators, like DFO, are able to promote angiogenesis ([60, 61]; and own observations), the Coll IV-stained blood vessels in the scar will confuse the quantification of the ECM Coll IV. A significant decrease in ECM Coll IV immunoreactivity was observed for BPY-DCA and DFO at all concentrations applied (Fig 8M and 8O). In relation to their respective controls, BPY-DCA led to an ECM collagen IV reduction of about 36%, while DFO showed a reducing effect of approximately 30%. The application of 100 μg/d cAMP also reduced Coll IV significantly in comparison to its control (Fig 8N). Application of 10 μg/d DFO for 2 weeks resulted in a similar extent of scar-reduction (data not shown).

### Effects of DFO on locomotor recovery

**Fig 9:**
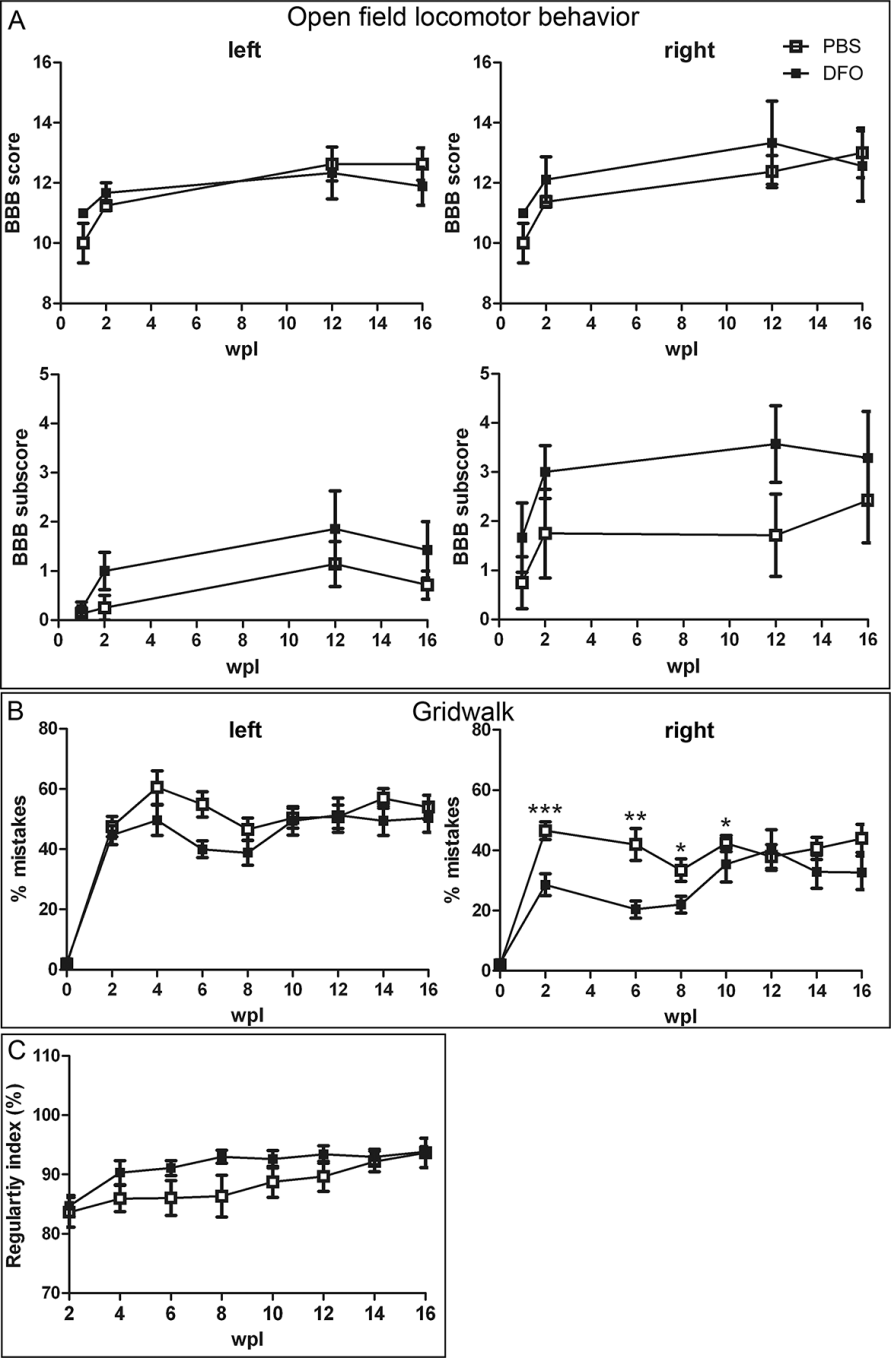
Effects of DFO on locomotor recovery. Long-term locomotor behavior in DFO-treated animals was assessed using the (A) BBB score and subscore, (B) Gridwalk and (C) Catwalk analysis. Left and right hindpaws were analyzed separately, because of asymmetry of the lesion, with the left rubrospinal tract being more affected than the right. Both BBB subscore and the Catwalk showed a positive but non-significant trend for DFO as compared to PBS controls. On the Gridwalk DFO-treated rats performed better than PBS controls. The percentage of mistakes of the right hindlimb decreased significantly during 2–10 weeks after injury, but was not significantly different from PBS controls during the last 4 weeks. Statistics: Mann-Whitney test * p < 0.05, ** p < 0.01, *** p< 0.001.

We applied 10 μg/d DFO for 2 weeks for a long-term effects behavioral study of 19 weeks. Locomotor recovery was evaluated over 1, 2, 12 and 16 weeks post-lesion using the open field locomotor BBB score and subscore. The horizontal ladder test (Gridwalk) and the Catwalk automated gait analysis were performed every second week. We evaluated the hindlimbs separately, because of a slight asymmetry in our Scouten wire knife lesion, where the right rubrospinal tract is less impaired than the left rubrospinal tract [10]. The BBB subscore showed a positive but non-significant trend for both paws in DFO-treated animals (Fig 9A). During the Gridwalk analysis the DFO-treated animals showed significantly less foot slips with the right hindlimb than the controls at 2, 6, 8 and 10 weeks. However, the later timepoints were not significantly different from the PBS control group (Fig 9B). A positive trend, but no significant difference was observed at 4 and 6 weeks for the left hindlimb. The Catwalk analysis revealed a positive but non-significant trend for DFO in the regularity index, a measure for limb coordination (Fig 9C). Similar to the Gridwalk, this trend disappeared at later timepoints.

### Long-term *in vivo* effects of DFO on scar reduction and axon regeneration

**Fig 10:**
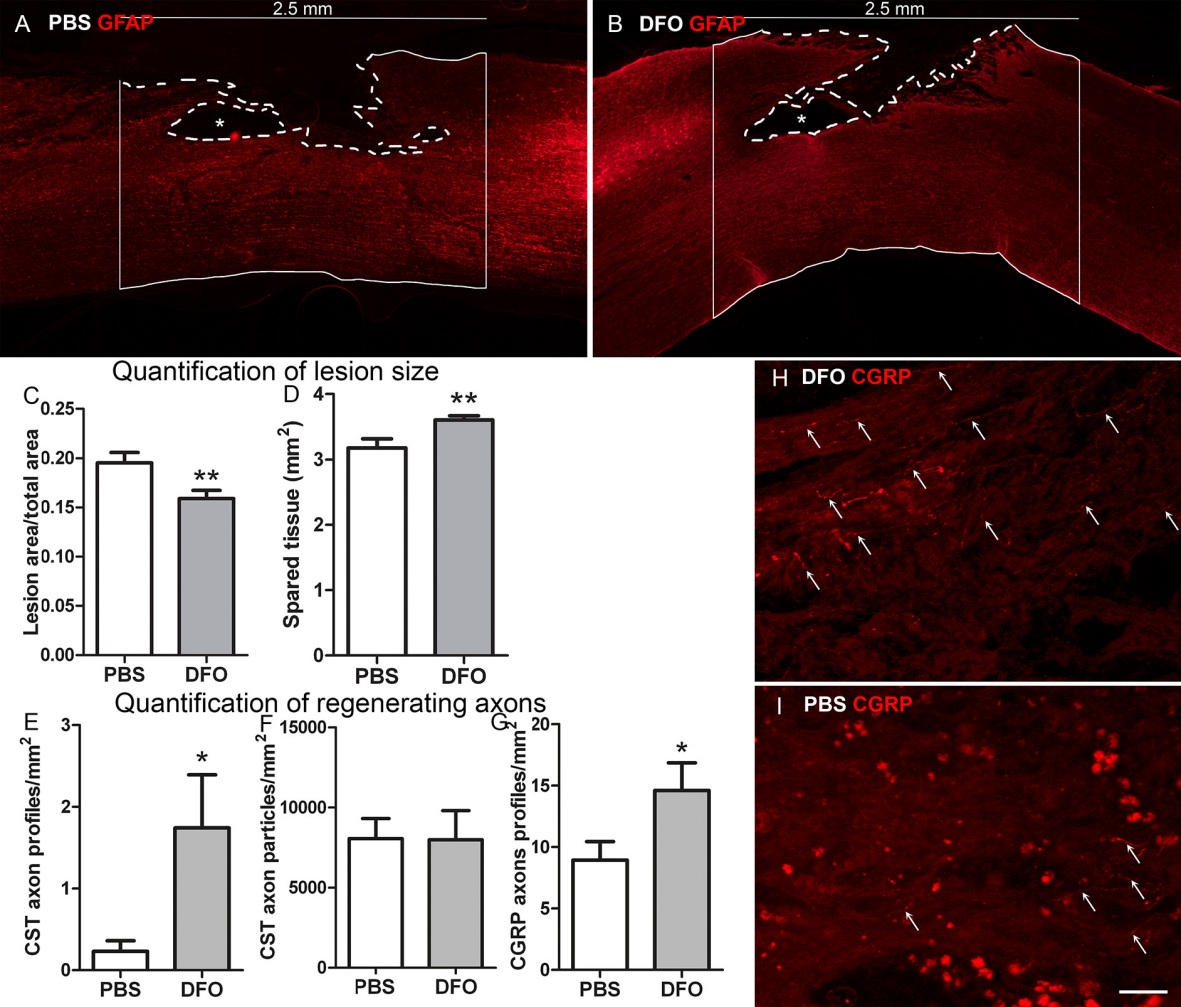
Effects of DFO on scarring and regeneration. Tissue preservation, lesion size and axon regeneration at 19 dpl. (A) Exemplary mosaic compositions of PBS- (A) and DFO- (B) treated spinal cord stained with anti-GFAP to visualize the GFAP-negative scar area. Lesion area marked with dotted lines, Asterisks indicate a cystic area in both treatment groups. S3 Fig provides pictures A and B with higher intensity to show that the lesion site is filled with tissue. Straight lines outline the region of interest used for quantification of lesion site (2.5 mm). DFO animals had significantly smaller lesion areas (corrected for the total area) than PBS controls (C) and significantly more tissue sparing (D) and. (E) Quantification of the number of CST axons per mm2 of lesion area revealed a significant increase. (F) the CST tracing was equal in both groups. (G) The number of CGRP axons in the scar was significantly increased in DFO-treated rats. (H, I) Representative pictures of CGRP axon profiles (arrows) in the lesion area in DFO- and PBS-treated animals. Note that the red spots in I originate from aberrantly stained macrophages. Statistics: unpaired T-test * p < 0.05, ** p < 0.01. Scale bar (G, H) = 50 μm.

At 19 weeks post-lesion, the animals were perfused and the spinal cords analyzed. By quantification of the GFAP-negative scar area, we found a significant reduction of the lesion size by DFO as compared to the PBS control (Fig 10A and 10C). Moreover, we observed that DFO-treated spinal cords in general were thicker around the lesion zone than PBS controls. Quantification of a 2.5 mm stretch of tissue around the lesion site (1.25 mm in each direction measured from the lesion centre), in sections where the central channel was included, revealed a DFO-induced increase in spared tissue (Fig 10D). Axon regeneration was studied in the same animals by counting BDA-traced descending CST axons and CGRP-stained ascending axons. A significant increase in the number of CGRP-positive axons profiles per mm^2^ of scar area was observed in DFO-treated animals as compared to controls (Fig 10G–10I). A small number of regenerating CST axons was found in the scar area, which was significantly increased in the DFO-treated animals (Fig 10E). There was no difference in the efficiency of CST tracing between the groups (Fig 10F).

## Discussion

### Clusters in the *in vitro* scarring model resemble the lesion scar in traumatic CNS injury

In order to study mechanisms of CNS lesion scarring as well as to develop new scar-modulating treatments, we modified the *in vitro* scarring model from Kimuro-Kuroda et al. (2010) [19]. The astroglial-meningeal fibroblast co-cultures result in a reproducible number of scar-like clusters at 7 days after stimulation with TGF-β. We observed that clusters were formed not only at the astrocyte-fibroblast border but also within the astrocyte-free fibroblast cell layer. At the fibroblast-astrocyte border, the glial cells surrounded the clusters while the fibroblasts populated the center, thus resembling the cellular arrangement seen in lesion scar *in vivo* [62]. We conclude that the formation of the fibrous scar is, at least in part, independent of the presence of astrocytes, although the latter seem to support the formation of clusters at the astrocyte-fibroblast border, an observation we made during the live imaging experiments. Indeed, meningeal fibroblasts and astrocytes cultured together resulted in differential gene expression as compared to the respective monocultures. This is illustrated by the TGF-induced downregulation of *Sema3A* and *EphB2* in the co-cultures but not in monocultures and the downregulation of *phosphacan* in astrocytes, which was not observed in the co-cultures. We conclude that the astrocytes and fibroblasts interact and are likely to secrete factors influencing each other.

Consistent with previous work [19], the clusters in our modified model displayed positive staining for the ECM molecules collagen IV and fibronectin, as well as the axon growth inhibitors NG-2, Sema3A, Tnc, Ephrin B2 and EphB2. In addition, we detected collagen I, III and V (F1C3), as well as neurocan and phosphacan. Importantly, the clusters contained molecules that were not (or only at very low levels) expressed by the fibroblasts, e.g. neurocan, phosphacan or Eph B2. This corroborates the hypothesis that the fibrous scar contains an extracellular matrix that could accumulate axon growth inhibitors secreted by surrounding astroglial cells. For example, it has been shown that Tnc (at the time of publication called J1-220) binds to collagen I-VI [63]. Although collagen is not inhibitory for axon outgrowth per se, reducing collagen deposition may diminish the amount of inhibitory components that bind to the collagenous scaffold of the ECM in fibrous scar [4, 64]. The here described TGF-β –induced upregulation of *Tnc*, *NG-2* and *neurocan* mRNA, as well as the lack of regulation of *phosphacan*, closely resemble the published regulation of these molecules at 7–8 days after *in vivo* spinal cord injury [2, 3]. This indicates that our *in vitro* model parallels the *in vivo* scar formation in view of timing of expression of ECM and growth inhibitory molecules.

### Cluster formation is reduced by DFO and cAMP

We investigated the mechanisms of scar-formation and found that the clusters were formed by proliferation as well as reorganization and contraction of the meningeal fibroblasts. The effects of the treatments on cluster formation are summarized in Table 3. Incubation of the TGF-β-stimulated co-cultures with DFO or cAMP led to a significant and consistent reduction of the number of scar-like clusters. In some cases, DFO did not diminish the number of clusters, but rather reduced the cluster size, resulting in a significant decline of the total cluster area. BPY-DCA, on the other hand, had no effect on cluster numbers or size and the effects of BPY-DCA and cAMP together were not significantly different to those of cAMP alone. Interestingly, DFO in combination with cAMP led to significantly less clusters than cAMP alone (or DFO alone), indicating an additional effect of DFO and cAMP. We conclude that from the iron chelators tested only DFO effectively reduced cluster formation. However, the treatment of spinal cord injury with BPY-DCA + cAMP *in vivo* led to a reduction in scar formation and an increase in axon regeneration, which was not observed with cAMP alone [8]. Due to the two negatively charged carboxylic acid groups BPY-DCA is cell-impermeable, as previously shown for related pyridine dicarboxylates and thus cannot perform its inhibitory actions on the intracellular prolyl-4-hydroxylase enzyme necessary for collagen IV biosynthesis [65]. This may be the reason why BPY-DCA has no effects on *in vitro* cluster formation. It has been shown before that DFO, despite of its low lipid-solubility, can slowly enter cells via pinocytosis [66–69] and thus gains access to intracellular iron [69, 70]. The differential cell-permeability could explain why DFO, but not BPY-DCA, reduces the formation of scar-like clusters *in vitro*. The reduction of cluster formation by cAMP could be explained by the attenuation of fibroblast proliferation in the co-cultures, which is in line with previous studies [12]. DFO, however, did not significantly reduce fibroblast proliferation. Instead, the reduction in cluster formation by DFO treatment is likely to be due to the observed reduction in ECM components.

**Table 3:** Summary of the treatment effects.

Treatment	Scar reduction	Gene expression	Protein expression	Proliferation	Neurite outgrowth
**DFO**	yes	*collagen IV* & NG-2 ↓	Tnc & collagen ↓	=	↑
**BPY-DCA**	no	no effects	no effects	n.d.	no effects
**cAMP**	yes	*Tnc*↓	Tnc ↓	↓	no effects
**DFO + cAMP**	yes^1^	*NG-2* ^1^ ↓, NC & PC ↑	Tnc & collagen^3^ ↓	=	no effects
**BPY-DCA + cAMP**	yes^2^	*no effects*	Tnc ↓^2^	n.d.	no effects

^1^ significantly different from cAMP (additional effect of DFO)

^2^ effect due to cAMP alone

^3^ effect due to DFO alone

n.d. = not done

### Cluster composition is altered by DFO and cAMP

A second mechanism to reduce inhibitory scarring may be based on changes in molecular composition of the ECM. The effect of the iron chelator and cAMP treatments on ECM composition and expression of axon growth inhibitory molecules are summarized in Table 3. Treatment with DFO resulted in a significant decrease in *Coll IV* mRNA. Since iron chelation is expected to be mainly functional at the post-translational level through inhibition of prolyl-hydroxylation [11] and assembly of thermo-stable collagen triple helices, a reduction of collagen expression at the mRNA level was not necessarily expected. At the protein level DFO reduced some of the polypeptide constituents of collagen I/III/V as detected by the F1C3 antibody. Although it is more relevant to address the coll IV protein levels, unfortunately, there are no antibodies available for the detection of coll IV by Western blotting. The reduction of *coll IV* mRNA and the reduction of other collagen types as detected by Western blot strongly suggest that DFO affects the collagen scaffold in the scar tissue. Both DFO and cAMP partially reduced Tnc on the protein level, although only cAMP reduced *Tnc* mRNA levels. The discrepancy between RNA and protein levels in the DFO-treated cultures could be explained by binding of Tnc to the collagen scaffold [63]. Thus, accumulation of Tnc protein in the scar is likely to be diminished when collagen biosynthesis is inhibited by the iron chelator DFO. In general, the effects of the treatments on the ECM proteins seem at first sight quite weak. This may be due to the fact that the cells have been in culture for 7–14 days before adding TGF-β and the scar-reducing treatments. The ECM molecules that these cells deposited before the treatment could have partially masked the effects of the treatments on the clusters that are formed during the following 7 days.

Regarding the CSPGs, *NG-2* mRNA levels were only reduced by DFO but not by cAMP. Interestingly, the expression of *neurocan* and *phosphacan* was upregulated by of DFO + cAMP in combination, but not by the individual components. This indicates that the combination of DFO with cAMP may cause adverse effects with respect to an increase of the inhibitory properties of the scar. While cAMP-induced upregulation of proteoglycan synthesis by fibroblasts has been described before [71], our data indicate that DFO, but not BPY-DCA, reduced mRNA and protein expression of some ECM and growth inhibitory molecules. Indeed, several studies suggest that DFO is able to affect transcription. DFO was shown to be neuroprotective through activation of hypoxia-inducible factor 1α (HIF-1α), a transcription factor responsible for the transcription of, e.g., vascular endothelial growth factor (VEGF), glucose transporters and erythropoietin [72]. This indicates another benefit of DFO as a treatment option for SCI. Moreover, iron depletion by DFO leads to upregulation of the growth arrest and DNA damage 45α (GADD 45α) gene in cell lines [73]. GADD 45α is an epigenetic regulator that is expressed during successful peripheral nerve regeneration [74]. This would be another potentially beneficial effect of DFO in SCI.

### DFO increases neurite outgrowth on scar-like clusters

We observed enhanced neurite growth of neonatal cortical neurons particularly on those clusters where astrocytes were present in the proximity of the neuron. Since astrocytes secrete both permissive and inhibitory molecules, it is not surprising that axons grew also in regions where astrocytes were present. Axons rarely crossed inhibitor-rich areas, but they did not avoid those either. Due to the functional dichotomy of axon growth modulating factors the latter may differentially act to inhibit or support axon growth, like, e.g., the alternatively spliced subtypes of Tnc [28]. Tnc can act growth-promoting when present diffusely in the ECM but inhibitory when deposited in a boundary [75]. Since the Tnc deposition at the border of the clusters is more like a boundary, we assume that Tnc may act inhibitory on neurite growth on the clusters. Similarly, CSPGs are well-known for their axon-growth inhibitory properties [76] but they can also promote neurite growth and neuronal survival [77–80].

The effect of a treatment on axon growth on the scar-like cell clusters is likely to depend on the balance between stimulation and inhibition and thus resembles the situation *in vivo* where growth-promoting and growth-inhibiting molecules are produced by the same cells [81].

Stimulation of neurite outgrowth by cAMP was expected, because of the known conditioning effects of cAMP on injured neurons *in vivo* [82, 83]. However, in our co-cultures cAMP only affected neurite outgrowth on the fibroblast layer, but had no effect on the length of neurites growing on clusters. Although DFO had no significant effect on neurite outgrowth on the cluster-free cell layers, indicating that DFO did not have direct effects on intrinsic neurite growth, it was the only treatment that significantly enhanced neurite growth on the scar-like clusters, probably by reducing the axon growth inhibitory properties of the latter.

### DFO decreases the collagenous scar *in vivo* but only partially induces regeneration

With the knowledge acquired in the *in vitro* study, we applied DFO, BPY-DCA or cAMP *in vivo* in a spinal cord hemisection model of the rat using concentrations that were roughly similar to those applied *in vitro*. To our surprise, not only DFO but also BPY-DCA reduced the amount of ECM Coll IV in the scar. While both concentrations of DFO and BPY-DCA significantly reduced collagenous scarring, only the higher dose of cAMP (100 μg/d) led to significant Coll IV reduction. BPY-DCA and cAMP have been shown before to be effective only when applied in combination [8]. From the present study, however, we conclude that the combination of the two compounds is not necessary for scar reduction. Interestingly, a discrepancy exists between the *in vitro* and *in vivo* data for BPY-DCA. Although BPY-DCA had no effect on the scar numbers, size, and Coll IV expression *in vitro* it significantly reduced ECM collagen IV deposition *in vivo*. As discussed above, this lack of effect in culture could be due to decreased membrane permeability because of the carboxyl groups [65]. *In vivo*, however, extracellular iron increases extensively after spinal cord injury, presumably as a result from iron-leaking cells damaged by the injury and haemorrhage followed by degradation of haemoglobin [84]. Free unbound iron is highly reactive and catalyzes harmful free radical reactions, assisting secondary damage. Thus, independent of cell permeability, BPY-DCA might function as an antioxidant by preventing the formation of free radicals by the Fenton reaction and the peroxidation of proteins and lipids. Another possibility is that lipid peroxidation by oxidative stress leads to permeabilization of the cell membrane, so that BPY-DCA possibly enters the cells in the *in vivo* spinal cord injury model. Although DFO has a low lipid solubility, it has been shown that this iron chelator can enter cells slowly via pinocytosis [66–69] and thus has access to intracellular iron when incubated for ≥ 24h [69, 70]. Since we infused DFO for 7 or even 14 days, DFO could have entered the cells and depleted the intracellular iron, leading to an inhibition of the collagen biosynthesis, which is in line with the observed reduction in ECM Coll IV. We moved on with the DFO treatment, because this was the most effective treatment *in vitro* at the level of scar-reduction, ECM reduction and neurite outgrowth stimulation. We infused DFO for 2 weeks in the lesioned spinal cord and performed behavioral tests of locomotion during 16 weeks and examined the spinal cord tissue immunohistologically at 19 weeks post-lesion. DFO lead to tissue sparring and reduced lesion size, therefore, it seems to attenuate the expansion of secondary tissue damage possibly due to antioxidant or neuroprotective actions. With respect to functional recovery after SCI, we observed an improved performance of DFO-treated rats on the Gridwalk during the first 10 weeks after treatment. Recently, it was reported that between 3 and 6 weeks after spinal cord injury in mice large quantities of iron, which were previously incorporated in macrophages by phagocytosis of red blood cells and tissue debris (e.g. iron-rich myelin), are released due to ongoing pathophysiological processes [85]. Most likely, this will cause a second wave of secondary spinal cord damage, leading to further impairment of axon regeneration and a deteriorated locomotor performance. Moreover, we found significantly more CGRP and CST axon profiles in the lesion site of DFO-treated animals than in the controls, but we could not find any fibers beyond the lesion site. Hence, a 2 week DFO infusion is probably not sufficient to promote long-term functional recovery in general, since reportedly DFO has a short half-life [86].It might be necessary to extend the treatment of spinal cord injured rats for at least 6 weeks to induce long-term functional recovery.

### Conclusions

The primary culture model for scar formation described here is highly suitable for *in vitro* screening of new potential therapeutic treatments to suppress scar formation in CNS trauma. Effects of treatments on the number of scar-like clusters, their molecular and cellular composition and their permissiveness for neurite growth can be studied fast and quantitatively. The *in vitro* model is particularly suitable for treatments that act intracellularly or both intra- and extracellularly. However, compounds that are unable to pass through the plasma membrane (like BPY-DCA) act only in the extracellular environment and may show false-negative results in this *in vitro* assay. Nevertheless, treatments that significantly affect scarring and ECM in our model system have a high chance to modulate scarring, ECM composition and axon growth *in vivo* as well. This provides a great alternative to *in vivo* studies which involve many animals and time consuming analyses. Using the *in vitro* model, investigators obtain information regarding the effective dosage and mechanisms of action of their drugs of interest. In this study, we showed that the iron chelator DFO most effectively reduced the fibrotic scar and its extracellular matrix, rendering it more permissive for axon growth. In this respect, DFO was superior to any of the other single or combinatorial treatments we have tested here. The application of DFO in the spinal cord dorsal hemisection model confirmed the scar-reducing capacities of DFO both at short term (1 wpl reduction of Coll IV) and at long term (tissue preservation and smaller scar volumes at 19 wpl). In conclusion, the *in vitro* model presented here is a unique and efficient tool for fast pre-screening of scar-reducing reagents for application in traumatic spinal cord injuries either in single or in combinatorial treatment paradigms.

## Supporting Information

S1 Fig Effects of TGF-β and treatments on low abundant mRNAs for axon growth inhibiting proteins. Effects of TGF-β on the mRNA expression of Ephrin B2, EphB2 and Sema3A in cortical astrocytes and meningeal fibroblasts cultured separately and in the co-cultures (A). Levels of each target mRNA were normalized to the housekeeping gene *cyclophilin*. Statistics: unpaired T-test * p < 0.05, ** p < 0.01. (B) Effects of scar-reducing treatments. Plotted are the levels of target mRNAs normalized to *cyclophilin*. All targets were equally expressed in TGF-treated and–untreated astrocyte and fibroblast monolayers. However, their expression changed after TGF-β treatment of the co-cultures: Ephrin B2 was downregulated, whereas EphB2 increased. Sema3A, which was already very low-abundant, was even more downregulated in the co-cultures after TGF stimulation. The treatments did not change the levels of all three target molecules.
(TIF)

S2 Fig Collagen IV levels in the *in vivo* lesion. Representative high magnification pictures of the Coll IV-positive scar area for (A) PBS corresponding to (B) DFO treatment, (C) PBS corresponding to (D) cAMP treatment and (E) Tris corresponding to (F) BPY-DCA treatment. A reduction of the Coll IV ECM signal outside of the blood vessels is visible for all treatments. Scale bar = 100 μm
(TIF)

S3 Fig Lesion area *in vivo* is not empty. Black and white version of Fig 10 picture A and B with exaggerated correction of levels to show that the lesion area is really filled with tissue except for the cystic areas marked with asterisks.
(TIF)

S1 Movie Live imaging of the co-cultures early after TGF. Pictures were taken from onward 10 h after TGF-β stimulation every 10^th^ min for 8 hours. Left side: fibroblasts. Right side: astrocytes. The fibroblast layer reorganizes to form clusters that contract over time.
(AVI)

S2 Movie Live imaging of the co-cultures 1 day after TGF. Pictures were taken from onward 24 h after TGF-β stimulation every 10^th^ min for 8 hours. Left side: fibroblasts. Right side: astrocytes. The clusters contract further and are pulled towards the fibroblast layer.
(MPG)

## References

[1] Camand E, Morel MP, Faissner A, Sotelo C, Dusart I. Long-term changes in the molecular composition of the glial scar and progressive increase of serotoninergic fibre sprouting after hemisection of the mouse spinal cord. Eur J Neurosci. 2004;20(5):1161–76. Epub 2004/09/03. 10.1111/j.1460-9568.2004.03558.xEJN3558 [pii]. . 15341588

[2] Jones LL, Margolis RU, Tuszynski MH. The chondroitin sulfate proteoglycans neurocan, brevican, phosphacan, and versican are differentially regulated following spinal cord injury. Experimental Neurology. 2003;182(2):399–411. 10.1016/s0014-4886(03)00087-6 12895450

[3] Tang X, Davies JE, Davies SJ. Changes in distribution, cell associations, and protein expression levels of NG2, neurocan, phosphacan, brevican, versican V2, and tenascin-C during acute to chronic maturation of spinal cord scar tissue. J Neurosci Res. 2003;71(3):427–44. 10.1002/jnr.10523 . 12526031

[4] Klapka N, Müller HW. Collagen matrix in spinal cord injury. J Neurotrauma. 2006;23(3–4):422–35. Epub 2006/04/25. 10.1089/neu.2006.23.422 . 16629627

[5] De Winter F, Oudega M, Lankhorst AJ, Hamers FP, Blits B, Ruitenberg MJ, et al Injury-induced class 3 semaphorin expression in the rat spinal cord. Exp Neurol. 2002;175(1):61–75. Epub 2002/05/16. doi: 10.1006/exnr.2002.7884 S0014488602978842 [pii]. . 12009760 10.1006/exnr.2002.7884

[6] Bundesen LQ, Scheel TA, Bregman BS, Kromer LF. Ephrin-B2 and EphB2 regulation of astrocyte-meningeal fibroblast interactions in response to spinal cord lesions in adult rats. J Neurosci. 2003;23(21):7789–800. Epub 2003/08/29. 23/21/7789 [pii]. . 12944508 10.1523/JNEUROSCI.23-21-07789.2003 PMC6740614

[7] Guth L, Zhang Z, Steward O. The unique histopathological responses of the injured spinal cord. Implications for neuroprotective therapy. Annals of the New York Academy of Sciences. 1999;890:366–84. . 10668443 10.1111/j.1749-6632.1999.tb08017.x

[8] Klapka N, Hermanns S, Straten G, Masanneck C, Duis S, Hamers FP, et al Suppression of fibrous scarring in spinal cord injury of rat promotes long-distance regeneration of corticospinal tract axons, rescue of primary motoneurons in somatosensory cortex and significant functional recovery. The European journal of neuroscience. 2005;22(12):3047–58. 10.1111/j.1460-9568.2005.04495.x . 16367771

[9] Stichel CC, Müller HW. Experimental strategies to promote axonal regeneration after traumatic central nervous system injury. Prog Neurobiol. 1998;56(2):119–48. Epub 1998/10/07. S0301-0082(98)00033-1 [pii]. . 9760698 10.1016/s0301-0082(98)00033-1

[10] Schiwy N, Brazda N, Müller HW. Enhanced regenerative axon growth of multiple fibre populations in traumatic spinal cord injury following scar-suppressing treatment. Eur J Neurosci. 2009;30(8):1544–53. Epub 2009/10/13. 10.1111/j.1460-9568.2009.06929.xEJN6929 [pii]. . 19817844

[11] Hales NJ, Beattie JF. Novel inhibitors of prolyl 4-hydroxylase. 5. The intriguing structure-activity relationships seen with 2,2'-bipyridine and its 5,5'-dicarboxylic acid derivatives. Journal of medicinal chemistry. 1993;36(24):3853–8. . 8254616 10.1021/jm00076a014

[12] Duncan MR, Frazier KS, Abramson S, Williams S, Klapper H, Huang X, et al Connective tissue growth factor mediates transforming growth factor beta-induced collagen synthesis: down-regulation by cAMP. FASEB journal: official publication of the Federation of American Societies for Experimental Biology. 1999;13(13):1774–86. . 10506580

[13] Tom VJ, Steinmetz MP, Miller JH, Doller CM, Silver J. Studies on the development and behavior of the dystrophic growth cone, the hallmark of regeneration failure, in an in vitro model of the glial scar and after spinal cord injury. J Neurosci. 2004;24(29):6531–9. 10.1523/JNEUROSCI.0994-04.2004 . 15269264 PMC6729861

[14] Jain A, Brady-Kalnay SM, Bellamkonda RV. Modulation of Rho GTPase activity alleviates chondroitin sulfate proteoglycan-dependent inhibition of neurite extension. J Neurosci Res. 2004;77(2):299–307. 10.1002/jnr.20161 . 15211597

[15] Bähr M, Schwab ME. Antibody that neutralizes myelin-associated inhibitors of axon growth does not interfere with recognition of target-specific guidance information by rat retinal axons. Journal of neurobiology. 1996;30(2):281–92. 10.1002/(SICI)1097-4695(199606)30:2&lt;281::AID-NEU9&gt;3.0.CO;2-1 . 8738756

[16] Adcock KH, Brown DJ, Shearer MC, Shewan D, Schachner M, Smith GM, et al Axon behaviour at Schwann cell—astrocyte boundaries: manipulation of axon signalling pathways and the neural adhesion molecule L1 can enable axons to cross. Eur J Neurosci. 2004;20(6):1425–35. 10.1111/j.1460-9568.2004.03573.x . 15355310

[17] Shearer MC, Niclou SP, Brown D, Asher RA, Holtmaat AJ, Levine JM, et al The astrocyte/meningeal cell interface is a barrier to neurite outgrowth which can be overcome by manipulation of inhibitory molecules or axonal signalling pathways. Mol Cell Neurosci. 2003;24(4):913–25. . 14697658 10.1016/j.mcn.2003.09.004

[18] Wanner IB, Deik A, Torres M, Rosendahl A, Neary JT, Lemmon VP, et al A new in vitro model of the glial scar inhibits axon growth. Glia. 2008;56(15):1691–709. Epub 2008/07/12. 10.1002/glia.20721 18618667 PMC3161731

[19] Kimura-Kuroda J, Teng X, Komuta Y, Yoshioka N, Sango K, Kawamura K, et al An in vitro model of the inhibition of axon growth in the lesion scar formed after central nervous system injury. Mol Cell Neurosci. 2010;43(2):177–87. Epub 2009/11/10. 10.1016/j.mcn.2009.10.008S1044-7431(09)00238-3 [pii]. . 19897043

[20] Leask A. Potential therapeutic targets for cardiac fibrosis: TGFbeta, angiotensin, endothelin, CCN2, and PDGF, partners in fibroblast activation. Circulation research. 2010;106(11):1675–80. 10.1161/CIRCRESAHA.110.217737 . 20538689

[21] Shi-wen X, Parapuram SK, Pala D, Chen Y, Carter DE, Eastwood M, et al Requirement of transforming growth factor beta-activated kinase 1 for transforming growth factor beta-induced alpha-smooth muscle actin expression and extracellular matrix contraction in fibroblasts. Arthritis and rheumatism. 2009;60(1):234–41. 10.1002/art.24223 . 19116914

[22] Ihn H. Autocrine TGF-beta signaling in the pathogenesis of systemic sclerosis. Journal of dermatological science. 2008;49(2):103–13. 10.1016/j.jdermsci.2007.05.014 . 17628443

[23] O'Brien MF, Lenke LG, Lou J, Bridwell KH, Joyce ME. Astrocyte response and transforming growth factor-beta localization in acute spinal cord injury. Spine (Phila Pa 1976). 1994;19(20):2321–9; discussion 30. . 7846578 10.1097/00007632-199410150-00012

[24] McTigue DM, Popovich PG, Morgan TE, Stokes BT. Localization of transforming growth factor-beta1 and receptor mRNA after experimental spinal cord injury. Exp Neurol. 2000;163(1):220–30. 10.1006/exnr.2000.7372 . 10785461

[25] Buss A, Pech K, Kakulas BA, Martin D, Schoenen J, Noth J, et al TGF-beta1 and TGF-beta2 expression after traumatic human spinal cord injury. Spinal Cord. 2008;46(5):364–71. 10.1038/sj.sc.3102148 . 18040277

[26] Wang X, Chen W, Liu W, Wu J, Shao Y, Zhang X. The role of thrombospondin-1 and transforming growth factor-beta after spinal cord injury in the rat. J Clin Neurosci. 2009;16(6):818–21. 10.1016/j.jocn.2008.09.014 . 19342245

[27] Komuta Y, Teng X, Yanagisawa H, Sango K, Kawamura K, Kawano H. Expression of transforming growth factor-beta receptors in meningeal fibroblasts of the injured mouse brain. Cell Mol Neurobiol. 2010;30(1):101–11. Epub 2009/08/05. 10.1007/s10571-009-9435-x . 19653094 PMC11498595

[28] Dobbertin A, Czvitkovich S, Theocharidis U, Garwood J, Andrews MR, Properzi F, et al Analysis of combinatorial variability reveals selective accumulation of the fibronectin type III domains B and D of tenascin-C in injured brain. Exp Neurol. 2010;225(1):60–73. Epub 2010/05/11. 10.1016/j.expneurol.2010.04.019S0014-4886(10)00156-1 [pii]. . 20451518

[29] Grimpe B, Silver J. A novel DNA enzyme reduces glycosaminoglycan chains in the glial scar and allows microtransplanted dorsal root ganglia axons to regenerate beyond lesions in the spinal cord. J Neurosci. 2004;24(6):1393–7. 10.1523/JNEUROSCI.4986-03.2004 . 14960611 PMC6730336

[30] Asher RA, Morgenstern DA, Fidler PS, Adcock KH, Oohira A, Braistead JE, et al Neurocan is upregulated in injured brain and in cytokine-treated astrocytes. J Neurosci. 2000;20(7):2427–38. . 10729323 10.1523/JNEUROSCI.20-07-02427.2000 PMC6772249

[31] Kohta M, Kohmura E, Yamashita T. Inhibition of TGF-beta1 promotes functional recovery after spinal cord injury. Neurosci Res. 2009;65(4):393–401. 10.1016/j.neures.2009.08.017 . 19744530

[32] Takeuchi M, Kamei N, Shinomiya R, Sunagawa T, Suzuki O, Kamoda H, et al Human platelet-rich plasma promotes axon growth in brain-spinal cord coculture. Neuroreport. 2012;23(12):712–6. 10.1097/WNR.0b013e3283567196 . 22750774

[33] Park SM, Jung JS, Jang MS, Kang KS, Kang SK. Transforming growth factor-beta1 regulates the fate of cultured spinal cord-derived neural progenitor cells. Cell proliferation. 2008;41(2):248–64. 10.1111/j.1365-2184.2008.00514.x . 18336470 PMC6496837

[34] Echeverry S, Shi XQ, Haw A, Liu H, Zhang ZW, Zhang J. Transforming growth factor-beta1 impairs neuropathic pain through pleiotropic effects. Molecular pain. 2009;5:16 10.1186/1744-8069-5-16 19327151 PMC2669449

[35] King VR, Phillips JB, Brown RA, Priestley JV. The effects of treatment with antibodies to transforming growth factor beta1 and beta2 following spinal cord damage in the adult rat. Neuroscience. 2004;126(1):173–83. 10.1016/j.neuroscience.2004.03.035 . 15145083

[36] Tyor WR, Avgeropoulos N, Ohlandt G, Hogan EL. Treatment of spinal cord impact injury in the rat with transforming growth factor-beta. J Neurol Sci. 2002;200(1–2):33–41. . 12127673 10.1016/s0022-510x(02)00113-2

[37] Moon LD, Fawcett JW. Reduction in CNS scar formation without concomitant increase in axon regeneration following treatment of adult rat brain with a combination of antibodies to TGFbeta1 and beta2. Eur J Neurosci. 2001;14(10):1667–77. . 11860461 10.1046/j.0953-816x.2001.01795.x

[38] Rathore KI, Redensek A, David S. Iron homeostasis in astrocytes and microglia is differentially regulated by TNF-alpha and TGF-beta1. Glia. 2012;60(5):738–50. 10.1002/glia.22303 . 22298416

[39] Schmalenbach C, Müller HW. Astroglia-neuron interactions that promote long-term neuronal survival. J Chem Neuroanat. 1993;6(4):229–37. . 8397922 10.1016/0891-0618(93)90044-5

[40] Schira J, Gasis M, Estrada V, Hendricks M, Schmitz C, Trapp T, et al Significant clinical, neuropathological and behavioural recovery from acute spinal cord trauma by transplantation of a well-defined somatic stem cell from human umbilical cord blood. Brain. 2012;135(Pt 2):431–46. Epub 2011/09/10. 10.1093/brain/awr222[pii]. . 21903726

[41] Heck N, Garwood J, Schutte K, Fawcett J, Faissner A. Astrocytes in culture express fibrillar collagen. Glia. 2003;41(4):382–92. 10.1002/glia.10184 . 12555205

[42] Heck N, Garwood J, Dobbertin A, Calco V, Sirko S, Mittmann T, et al Evidence for distinct leptomeningeal cell-dependent paracrine and EGF-linked autocrine regulatory pathways for suppression of fibrillar collagens in astrocytes. Mol Cell Neurosci. 2007;36(1):71–85. 10.1016/j.mcn.2007.06.002 . 17689979

[43] Faissner A, Kruse J. J1/tenascin is a repulsive substrate for central nervous system neurons. Neuron. 1990;5(5):627–37. . 1699568 10.1016/0896-6273(90)90217-4

[44] Kruse F, Bosse F, Vogelaar CF, Brazda N, Kury P, Gasis M, et al Cortical gene expression in spinal cord injury and repair: insight into the functional complexity of the neural regeneration program. Frontiers in molecular neuroscience. 2011;4:26 10.3389/fnmol.2011.00026 21994489 PMC3182759

[45] Vogelaar CF, Gervasi NM, Gumy LF, Story DJ, Raha-Chowdhury R, Leung KM, et al Axonal mRNAs: characterisation and role in the growth and regeneration of dorsal root ganglion axons and growth cones. Mol Cell Neurosci. 2009;42(2):102–15. 10.1016/j.mcn.2009.06.002 . 19520167 PMC4603359

[46] Vogelaar CF, Hoekman MF, Brakkee JH, Bogerd J, Burbach JP. Developmental regulation of homeobox gene expression in dorsal root ganglion neurons is not recapitulated during regeneration of the crushed sciatic nerve. Neuroscience. 2004;125(3):645–50. 10.1016/j.neuroscience.2004.02.006 . 15099678

[47] Steward O, Zheng B, Tessier-Lavigne M. False resurrections: distinguishing regenerated from spared axons in the injured central nervous system. J Comp Neurol. 2003;459(1):1–8. 10.1002/cne.10593 . 12629662

[48] Iannotti CA, Clark M, Horn KP, van Rooijen N, Silver J, Steinmetz MP. A combination immunomodulatory treatment promotes neuroprotection and locomotor recovery after contusion SCI. Exp Neurol. 2011;230(1):3–15. 10.1016/j.expneurol.2010.03.010 . 20338167

[49] Basso DM, Beattie MS, Bresnahan JC. A sensitive and reliable locomotor rating scale for open field testing in rats. J Neurotrauma. 1995;12(1):1–21. . 7783230 10.1089/neu.1995.12.1

[50] Jeong MA, Plunet W, Streijger F, Lee JH, Plemel JR, Park S, et al Intermittent fasting improves functional recovery after rat thoracic contusion spinal cord injury. J Neurotrauma. 2011;28(3):479–92. 10.1089/neu.2010.1609 21219083 PMC3119327

[51] Lankhorst AJ, Duis SE, ter Laak MP, Joosten EA, Hamers FP, Gispen WH. Functional recovery after central infusion of alpha-melanocyte-stimulating hormone in rats with spinal cord contusion injury. Journal of neurotrauma. 1999;16(4):323–31. . 10225218 10.1089/neu.1999.16.323

[52] Kozlov AK, Kardamakis AA, Hellgren Kotaleski J, Grillner S. Gating of steering signals through phasic modulation of reticulospinal neurons during locomotion. Proc Natl Acad Sci U S A. 2014;111(9):3591–6. 10.1073/pnas.1401459111 24550483 PMC3948313

[53] Jordan LM. Initiation of locomotion in mammals. Annals of the New York Academy of Sciences. 1998;860:83–93. . 9928303 10.1111/j.1749-6632.1998.tb09040.x

[54] Metz GA, Whishaw IQ. The ladder rung walking task: a scoring system and its practical application. Journal of visualized experiments: JoVE. 2009;(28). 10.3791/1204 19525918 PMC2796662

[55] Metz GA, Whishaw IQ. Cortical and subcortical lesions impair skilled walking in the ladder rung walking test: a new task to evaluate fore- and hindlimb stepping, placing, and co-ordination. J Neurosci Methods. 2002;115(2):169–79. . 11992668 10.1016/s0165-0270(02)00012-2

[56] Hamers FP, Lankhorst AJ, van Laar TJ, Veldhuis WB, Gispen WH. Automated quantitative gait analysis during overground locomotion in the rat: its application to spinal cord contusion and transection injuries. J Neurotrauma. 2001;18(2):187–201. 10.1089/08977150150502613 . 11229711

[57] Lai YL, Smith PM, Lamm WJ, Hildebrandt J. Sampling and analysis of cerebrospinal fluid for chronic studies in awake rats. Journal of applied physiology: respiratory, environmental and exercise physiology. 1983;54(6):1754–7. . 6409862 10.1152/jappl.1983.54.6.1754

[58] Shellswell GB, Restall DJ, Duance VC, Bailey AJ. Identification and differential distribution of collagen types in the central and peripheral nervous systems. FEBS letters. 1979;106(2):305–8. . 387447 10.1016/0014-5793(79)80520-7

[59] Azzi G, Jouis V, Godeau G, Groult N, Robert AM. Immunolocalisation of extracellular matrix macromolecules in the rat spinal cord. Matrix. 1989;9(6):479–85. . 2635761 10.1016/s0934-8832(11)80017-x

[60] Beerepoot LV, Shima DT, Kuroki M, Yeo KT, Voest EE. Up-regulation of vascular endothelial growth factor production by iron chelators. Cancer Res. 1996;56(16):3747–51. . 8706019

[61] Ikeda Y, Tajima S, Yoshida S, Yamano N, Kihira Y, Ishizawa K, et al Deferoxamine promotes angiogenesis via the activation of vascular endothelial cell function. Atherosclerosis. 2011;215(2):339–47. 10.1016/j.atherosclerosis.2011.01.009 . 21315355

[62] Brazda N, Müller HW. Pharmacological modification of the extracellular matrix to promote regeneration of the injured brain and spinal cord. Prog Brain Res. 2009;175:269–81. Epub 2009/08/08. 10.1016/S0079-6123(09)17518-0S0079-6123(09)17518-0 [pii]. . 19660662

[63] Faissner A, Kruse J, Kuhn K, Schachner M. Binding of the J1 adhesion molecules to extracellular matrix constituents. J Neurochem. 1990;54(3):1004–15. . 2303805 10.1111/j.1471-4159.1990.tb02350.x

[64] Fawcett JW, Schwab ME, Montani L, Brazda N, Müller HW. Defeating inhibition of regeneration by scar and myelin components. Handb Clin Neurol. 2012;109:503–22. Epub 2012/10/27. 10.1016/B978-0-444-52137-8.00031-0B978-0-444-52137-8.00031-0 [pii]. . 23098733

[65] Tschank G, Raghunath M, Gunzler V, Hanauske-Abel HM. Pyridinedicarboxylates, the first mechanism-derived inhibitors for prolyl 4-hydroxylase, selectively suppress cellular hydroxyprolyl biosynthesis. Decrease in interstitial collagen and Clq secretion in cell culture. The Biochemical journal. 1987;248(3):625–33. 2829835 10.1042/bj2480625 PMC1148595

[66] Lloyd JB, Cable H, Rice-Evans C. Evidence that desferrioxamine cannot enter cells by passive diffusion. Biochem Pharmacol. 1991;41(9):1361–3. . 2018567 10.1016/0006-2952(91)90109-i

[67] Palmer C, Roberts RL, Bero C. Deferoxamine posttreatment reduces ischemic brain injury in neonatal rats. Stroke; a journal of cerebral circulation. 1994;25(5):1039–45. . 8165675 10.1161/01.str.25.5.1039

[68] Samuni Y, Coffin D, DeLuca AM, DeGraff WG, Venson DJ, Ambudkar I, et al The use of Zn-desferrioxamine for radioprotection in mice, tissue culture, and isolated DNA. Cancer Res. 1999;59(2):405–9. . 9927054

[69] Simonart T, Degraef C, Andrei G, Mosselmans R, Hermans P, Van Vooren JP, et al Iron chelators inhibit the growth and induce the apoptosis of Kaposi's sarcoma cells and of their putative endothelial precursors. The Journal of investigative dermatology. 2000;115(5):893–900. 10.1046/j.1523-1747.2000.00119.x . 11069629

[70] Kicic A, Chua AC, Baker E. Effect of iron chelators on proliferation and iron uptake in hepatoma cells. Cancer. 2001;92(12):3093–110. . 11753989 10.1002/1097-0142(20011215)92:12<3093::aid-cncr10107>3.0.co;2-b

[71] Imai Y, Ibaraki K, Odajima R, Shishiba Y. Effects of dibutyryl cyclic AMP on hyaluronan and proteoglycan synthesis by retroocular tissue fibroblasts in culture. Endocrine journal. 1994;41(6):645–54. . 7704088 10.1507/endocrj.41.645

[72] Hamrick SE, McQuillen PS, Jiang X, Mu D, Madan A, Ferriero DM. A role for hypoxia-inducible factor-1alpha in desferoxamine neuroprotection. Neurosci Lett. 2005;379(2):96–100. 10.1016/j.neulet.2004.12.080 . 15823423

[73] Saletta F, Suryo Rahmanto Y, Siafakas AR, Richardson DR. Cellular iron depletion and the mechanisms involved in the iron-dependent regulation of the growth arrest and DNA damage family of genes. J Biol Chem. 2011;286(41):35396–406. 10.1074/jbc.M111.273060 21852233 PMC3195607

[74] Befort K, Karchewski L, Lanoue C, Woolf CJ. Selective up-regulation of the growth arrest DNA damage-inducible gene Gadd45 alpha in sensory and motor neurons after peripheral nerve injury. Eur J Neurosci. 2003;18(4):911–22. . 12925017 10.1046/j.1460-9568.2003.02827.x

[75] Deller T, Haas CA, Naumann T, Joester A, Faissner A, Frotscher M. Up-regulation of astrocyte-derived tenascin-C correlates with neurite outgrowth in the rat dentate gyrus after unilateral entorhinal cortex lesion. Neuroscience. 1997;81(3):829–46. Epub 1997/10/08. S0306-4522(97)00194-2 [pii]. . 9316032 10.1016/s0306-4522(97)00194-2

[76] Fitch MT, Silver J. CNS injury, glial scars, and inflammation: Inhibitory extracellular matrices and regeneration failure. Exp Neurol. 2008;209(2):294–301. 10.1016/j.expneurol.2007.05.014 17617407 PMC2268907

[77] Junghans U, Koops A, Westmeyer A, Kappler J, Meyer HE, Müller HW. Purification of a meningeal cell-derived chondroitin sulphate proteoglycan with neurotrophic activity for brain neurons and its identification as biglycan. Eur J Neurosci. 1995;7(11):2341–50. . 8563983 10.1111/j.1460-9568.1995.tb00655.x

[78] Kappler J, Junghans U, Koops A, Stichel CC, Hausser HJ, Kresse H, et al Chondroitin/dermatan sulphate promotes the survival of neurons from rat embryonic neocortex. Eur J Neurosci. 1997;9(2):306–18. . 9058051 10.1111/j.1460-9568.1997.tb01401.x

[79] Koops A, Kappler J, Junghans U, Kuhn G, Kresse H, Müller HW. Cultured astrocytes express biglycan, a chondroitin/dermatan sulfate proteoglycan supporting the survival of neocortical neurons. Brain Res Mol Brain Res. 1996;41(1–2):65–73. . 8883935 10.1016/0169-328x(96)00067-8

[80] Faissner A, Clement A, Lochter A, Streit A, Mandl C, Schachner M. Isolation of a neural chondroitin sulfate proteoglycan with neurite outgrowth promoting properties. J Cell Biol. 1994;126(3):783–99. 7519189 10.1083/jcb.126.3.783 PMC2120143

[81] Jones LL, Sajed D, Tuszynski MH. Axonal regeneration through regions of chondroitin sulfate proteoglycan deposition after spinal cord injury: a balance of permissiveness and inhibition. J Neurosci. 2003;23(28):9276–88. Epub 2003/10/17. 23/28/9276 [pii]. . 14561854 10.1523/JNEUROSCI.23-28-09276.2003 PMC6740563

[82] Hannila SS, Filbin MT. The role of cyclic AMP signaling in promoting axonal regeneration after spinal cord injury. Exp Neurol. 2008;209(2):321–32. 10.1016/j.expneurol.2007.06.020 17720160 PMC2692909

[83] Qiu J, Cai D, Dai H, McAtee M, Hoffman PN, Bregman BS, et al Spinal axon regeneration induced by elevation of cyclic AMP. Neuron. 2002;34(6):895–903. . 12086638 10.1016/s0896-6273(02)00730-4

[84] Moos T, Morgan EH. The metabolism of neuronal iron and its pathogenic role in neurological disease: review. Annals of the New York Academy of Sciences. 2004;1012:14–26. . 15105252 10.1196/annals.1306.002

[85] Rathore KI, Kerr BJ, Redensek A, Lopez-Vales R, Jeong SY, Ponka P, et al Ceruloplasmin protects injured spinal cord from iron-mediated oxidative damage. J Neurosci. 2008;28(48):12736–47. 10.1523/JNEUROSCI.3649-08.2008 . 19036966 PMC6671786

[86] Howland MA. Risks of parenteral deferoxamine for acute iron poisoning. Journal of toxicology Clinical toxicology. 1996;34(5):491–7. . 8800186 10.3109/15563659609028006

