# Supplementary figures and images for: Pharmacological Suppression of CNS Scarring by Deferoxamine Reduces Lesion Volume and Increases Regeneration in an In Vitro Model for Astroglial-Fibrotic Scarring and in Rat Spinal Cord Injury In Vivo
Source: PLoS One. 2015 Jul 29;10(7):e0134371. doi: 10.1371/journal.pone.0134371 (PMC4519270; doi:10.1371/journal.pone.0134371)

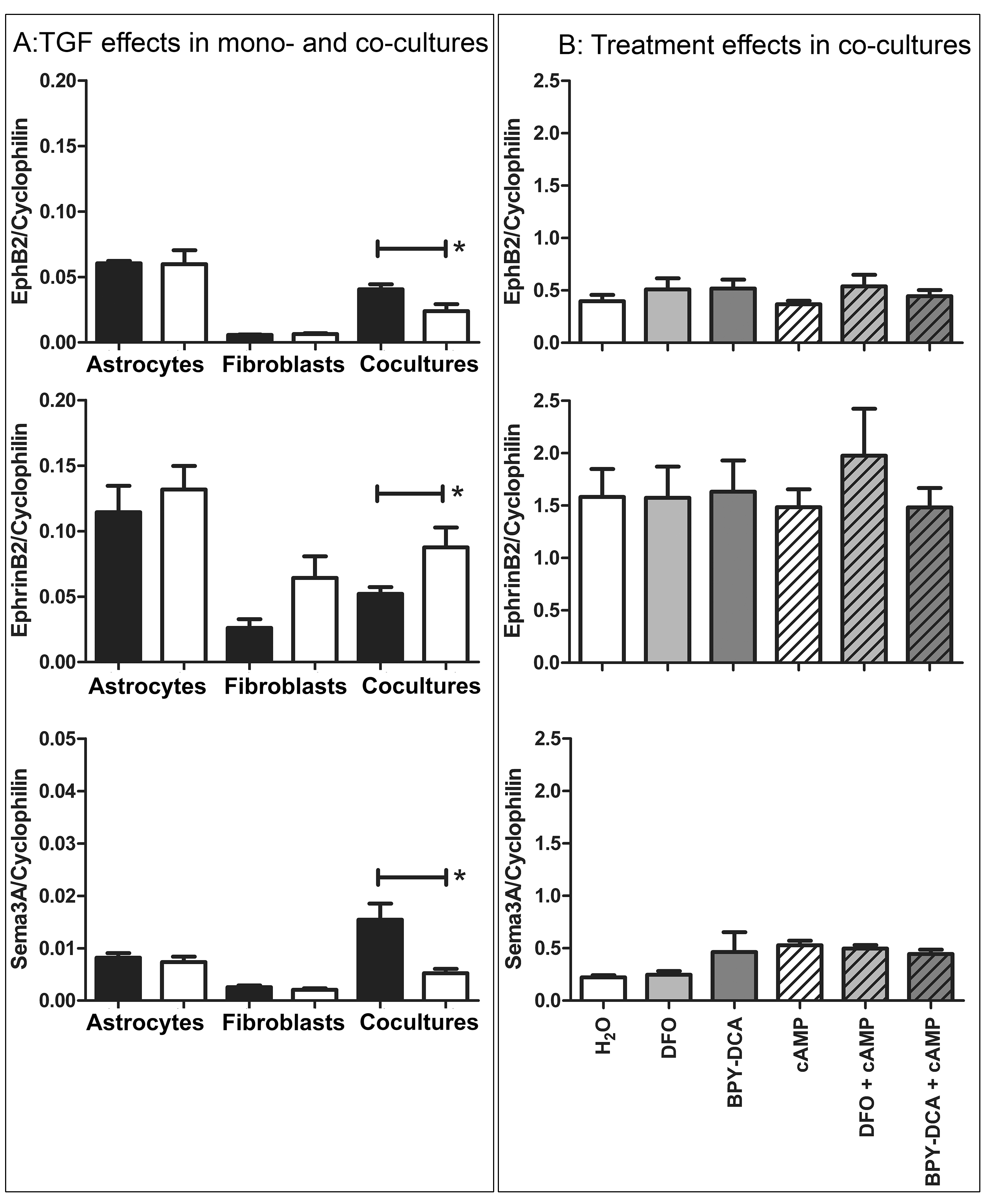

Supplement: S1 Fig — Effects of TGF-β on the mRNA expression of Ephrin B2, EphB2 and Sema3A in cortical astrocytes and meningeal fibroblasts cultured separately and in the co-cultures (A). Levels of each target mRNA were normalized to the housekeeping gene cyclophilin. Statistics: unpaired T-test * p < 0.05, ** p < 0.01. (B) Effects of scar-reducing treatments. Plotted are the levels of target mRNAs normalized to cyclophilin. All targets were equally expressed in TGF-treated and–untreated astrocyte and fibroblast monolayers. However, their expression changed after TGF-β treatment of the co-cultures: Ephrin B2 was downregulated, whereas EphB2 increased. Sema3A, which was already very low-abundant, was even more downregulated in the co-cultures after TGF stimulation. The treatments did not change the levels of all three target molecules. (TIF) [file pone.0134371.s001.tif]

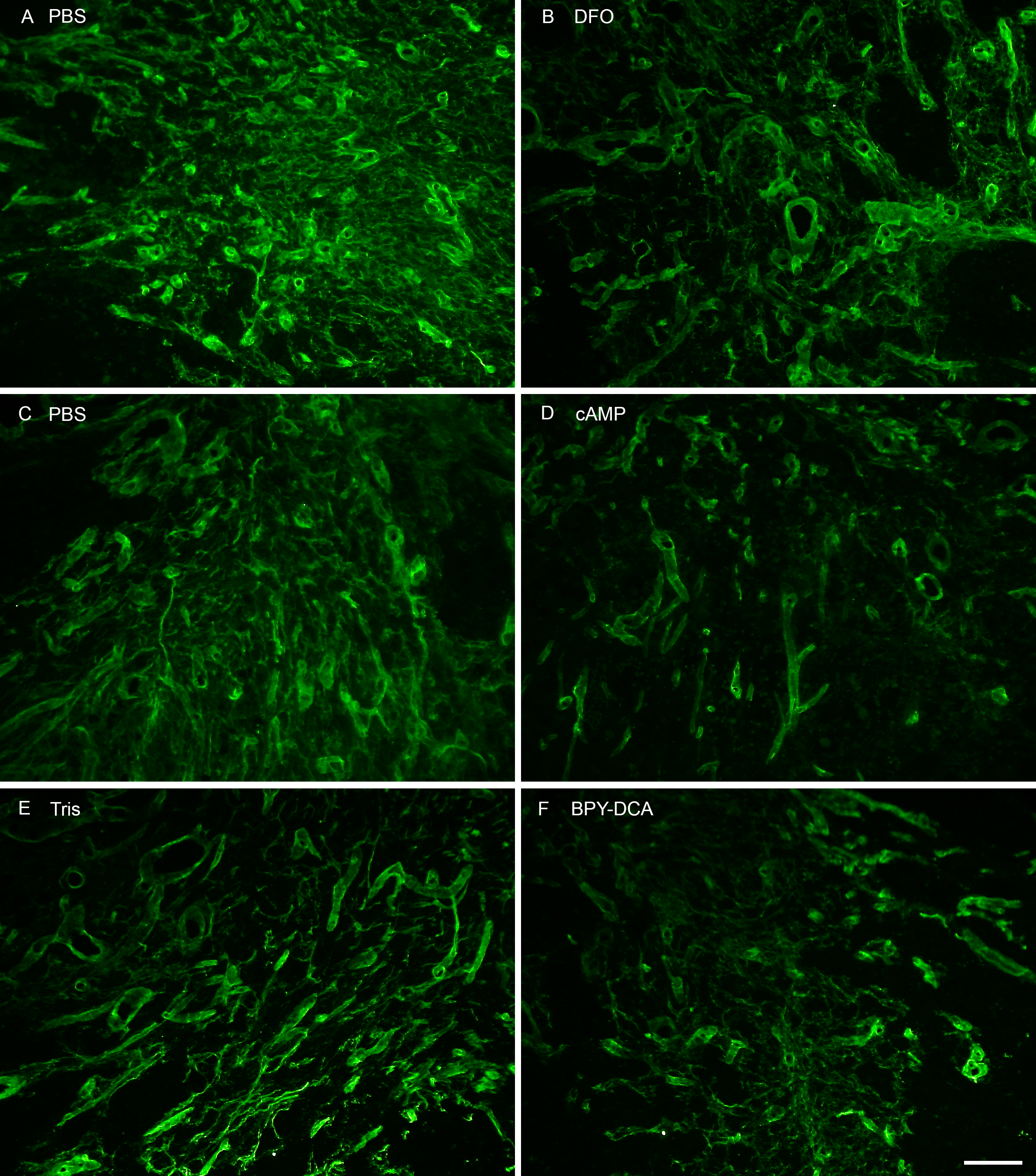

Supplement: S2 Fig — Representative high magnification pictures of the Coll IV-positive scar area for (A) PBS corresponding to (B) DFO treatment, (C) PBS corresponding to (D) cAMP treatment and (E) Tris corresponding to (F) BPY-DCA treatment. A reduction of the Coll IV ECM signal outside of the blood vessels is visible for all treatments. Scale bar = 100 μm (TIF) [file pone.0134371.s002.tif]

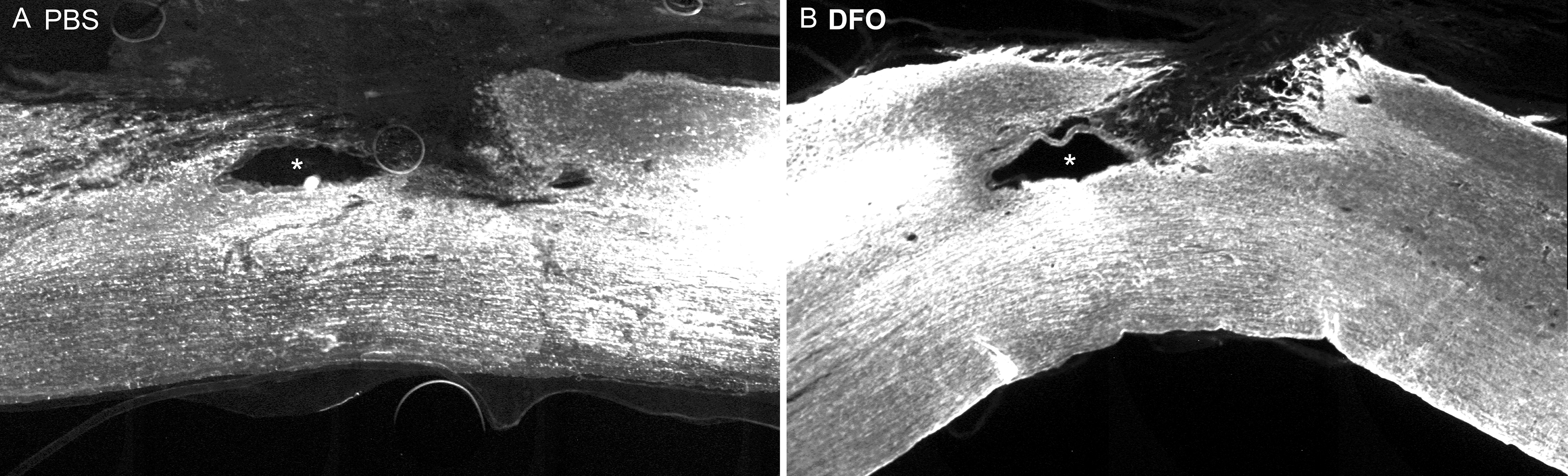

Supplement: S3 Fig — Black and white version of Fig 10 picture A and B with exaggerated correction of levels to show that the lesion area is really filled with tissue except for the cystic areas marked with asterisks. (TIF) [file pone.0134371.s003.tif]
